# A novel streptococcal integrative conjugative element involved in iron acquisition

**DOI:** 10.1111/j.1365-2958.2008.06481.x

**Published:** 2008-10-22

**Authors:** Zoe Heather, Matthew T G Holden, Karen F Steward, Julian Parkhill, Lijiang Song, Gregory L Challis, Carl Robinson, Nicholas Davis-Poynter, Andrew S Waller

**Affiliations:** 1 Centre for Preventive Medicine, Animal Health TrustLanwades Park, Kentford, Newmarket, Suffolk CB8 7UU, UK; 2 Wellcome Trust Sanger Institute, Wellcome Trust Genome CampusHinxton, Cambridge CB10 1SA, UK; 3 Department of Chemistry, University of WarwickCoventry CV4 7AL, UK

## Abstract

In this study, we determined the function of a novel non-ribosomal peptide synthetase (NRPS) system carried by a streptococcal integrative conjugative element (ICE), ICE*Se2*. The NRPS shares similarity with the yersiniabactin system found in the high-pathogenicity island of *Yersinia* sp. and is the first of its kind to be identified in streptococci. We named the NRPS product ‘equibactin’ and genes of this locus *eqbA–N.* ICE*Se2*, although absolutely conserved in *Streptococcus equi*, the causative agent of equine strangles, was absent from all strains of the closely related opportunistic pathogen *Streptococcus zooepidemicus*. Binding of EqbA, a DtxR-like regulator, to the *eqbB* promoter was increased in the presence of cations. Deletion of *eqbA* resulted in a small-colony phenotype. Further deletion of the *irp2* homologue *eqbE*, or the genes *eqbH*, *eqbI* and *eqbJ* encoding a putative ABC transporter, or addition of the iron chelator nitrilotriacetate, reversed this phenotype, implicating iron toxicity. Quantification of ^55^Fe accumulation and sensitivity to streptonigrin suggested that equibactin is secreted by *S. equi* and that the *eqbH*, *eqbI* and *eqbJ* genes are required for its associated iron import. In agreement with a structure-based model of equibactin synthesis, supplementation of chemically defined media with salicylate was required for equibactin production.

## Introduction

Horizontal gene transfer has facilitated greatly microbial evolution and rapid adaptation to new niches. The term ICE has been adopted to describe all self-transmissible mobile genetic elements with predicted or demonstrated integrative and conjugative properties (Burrus *et al*., 2002a, b). ICEs excise from the donor chromosome to form a non-replicative circular molecule, then promote self-transfer to a new host by conjugation and subsequently insert into a new target site. This transfer can occur between genetically unrelated bacteria.

Tn*916*, identified in *Enterococcus faecalis*, was the first ICE to be completely sequenced (Flannagan *et al*., 1994). Recently, the number of sequenced ICEs has risen considerably, particularly with the advent of genome sequencing (Burrus *et al*., 2002a, b; Sebaihia *et al*., 2006). Comparisons between ICEs of low G+C Gram-positive bacteria have revealed that these elements are widespread and possess a modular organization (Garnier *et al*., 2000; Roberts *et al*., 2001; Burrus *et al*., 2002b). Each of these modules includes all of the coding sequences required for a specific biological function, namely site-specific recombination, conjugation and regulation. This is not dissimilar to the combination of functional modules seen in other bacterial mobile elements such as phages and plasmids (Toussaint and Merlin, 2002) and allows these structures, even when unrelated or distantly related, to evolve by exchange of modules (Burrus *et al*., 2002b). Consequently, a diverse set of mosaic ICEs are emerging; most encode a tyrosine recombinase (like the majority of integrated prophage) but a small number have been identified that utilize a serine recombinase to mediate site-specific recombination (Wang *et al*., 2000; Burrus *et al*., 2002a).

ICEs have played an important role in the distribution of antibiotic resistance loci. Other functions encoded by ICEs include toxic aromatic compound degradation pathways, sucrose utilization, bacteriocin biosynthesis, heavy metal resistance, type III and type IV secretion systems, capsule antigen and a metalloprotease virulence factor (Burrus and Waldor, 2004; Franco, 2004). A novel ICE in the ECOR31 strain of *Escherichia coli* carries the high-pathogenicity island (HPI) encoding the yersiniabactin siderophore system, which is critical to the virulence of *Yersinia* sp. and is widely distributed among *E. coli* strains and other *Enterobacteriaceae* that cause extraintestinal infections (Schubert *et al*., 2004).

The acquisition of iron is an essential process for all pathogenic bacteria. In mammalian host tissue iron is sequestered by transferrin, lactoferrin or in red blood cells (Wooldridge and Williams, 1993). Bacterial pathogens access this limited iron supply through the production of cell surface receptors for transferrin and lactoferrin, utilizing haem-containing compounds, iron transporters, or by the synthesis and secretion of iron-sequestering siderophores (Wandersman and Delepelaire, 2004). *Streptococcus pneumoniae* encodes multiple iron transporters that are important for virulence (Brown *et al*., 2002) and a cell-surface lactoferrin receptor (Hakansson *et al*., 2001). *Streptococcus pyogenes* utilizes haemoprotein binding and transport proteins (Lei *et al*., 2002) and a multimetal transport system (Janulczyk *et al*., 2003). *Streptococcus mutans* transports ferrous iron and produces a Mn/Fe uptake system that is important *in vivo* (Evans *et al*., 1986). Recently, a siderophore-iron(III) transport gene was identified in *S. pyogenes*, *S. agalactiae* and *S. pneumoniae* (Hanks *et al*., 2005; Clancy *et al*., 2006; Pramanik and Braun, 2006), but siderophore biosynthesis has not been previously identified in any streptococci (Eichenbaum *et al*., 1996).

*Streptococcus equi* is the causative agent of strangles, one of the most commonly diagnosed and important infectious diseases of horses worldwide. The disease is characterized by pyrexia followed by profuse nasal discharge and abscessation of the lymph nodes of the head and neck. These can restrict the airway and it is this clinical feature that gave the disease ‘strangles’ its name. Approximately 10% of horses that recover from strangles become persistently infected and may intermittently shed *S. equi* for several years. This ‘carrier state’ is believed to be fundamental to the epidemiology of strangles and originates from incomplete drainage of the guttural pouches and/or sinuses following rupture of abscesses formed in the retropharyngeal lymph nodes (Newton *et al*., 1997; 2000; Waller and Jolley, 2007).

Genome sequencing of *S. equi* and its close ancestral relative *Streptococcus zooepidemicus* has enabled us to identify possible virulence determinants in the former that could contribute to its increased pathogenicity (data to be reported elsewhere). In this study, our aim was to determine the function of a yersiniabactin-like non-ribosomal peptide synthetase (NRPS) system encoded by a novel ICE, ICE*Se2* identified in *S. equi*. Although we have not yet identified the peptide made by this NRPS, data consistent with its role in iron acquisition have been established. This is the first example of a NRPS system involved in the acquisition of iron to be identified in streptococci.

## Results

### Identification and prevalence of a novel ICE encoding a siderophore-like NRPS in *S. equi*

The genomes of *S. equi* 4047 and *S. zooepidemicus* H70 share > 97% DNA sequence identity although their *in vivo* virulence differs markedly. Comparison of the *S. equi* 4047 genome and *S. zooepidemicus* H70 unfinished genome using the Artemis Comparison Tool (Carver *et al*., 2005) led to the identification of a large 63 kb locus in *S. equi* 4047 that was absent from the *S. zooepidemicus* H70 genome, had a significant decrease in G+C content (31% compared with 42% across the whole *S. equi* genome) and was bordered by 108 bp perfect inverted repeat elements.

blastp and fasta analysis against UniProt determined that the locus contained coding regions with similarity and conserved gene order with coding sequences (CDSs) contained within the conjugative transposons CDTn*2* and CDTn*5* in the genome of *Clostridium difficile* strain 630 (Sebaihia *et al*., 2006) and the plasmid-borne Tn*1549* in *Enterococcus* spp. (Garnier *et al*., 2000) (Fig. S1). Consequently we have named this *S. equi* integrative conjugative element, ICE*Se2*, according to the suggested nomenclature (Burrus *et al*., 2002a). Like Tn*916*, these elements appear modular, with CDSs likely to be associated with conjugation and site-specific recombination located in the left and right extremities respectively. The recombination module of ICE*Se2* encodes a single serine recombinase [284 amino acids (aa)] and has homology (43–47% aa sequence identity) to the N-terminal regions (first 277–303 aa) of similar proteins in CDTn*2* and CDTn*5* and also TndX (533 aa, 43% aa sequence identity), which is responsible for the integration and excision of the conjugative transposon Tn*5397* (Wang *et al*., 2000). A closer homologue is also present in the genome of *S. pyogenes* strain MGAS10750 (557 aa, 64% aa sequence identity). The sequence and annotation of ICE*Se2* has been submitted to GenBank/EMBL with the Accession No. AM909652.

**Fig. 1:**
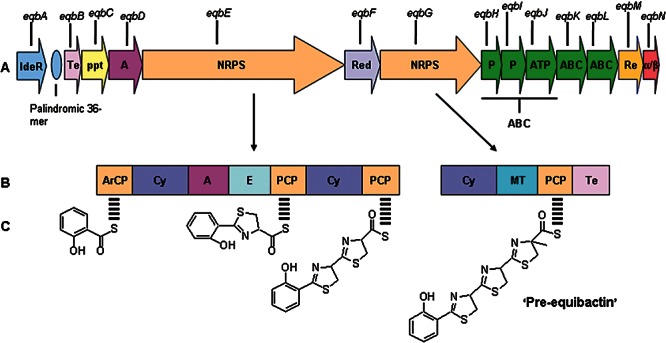
The equibactin locus and predicted functions of the *eqb* gene products. A. Predicted functions of CDSs in the ICE*Se2 eqb* cluster [IdeR, iron-dependent regulator; Te, type II thioesterase; ppt, 4′-phosphopantetheinyl transferase; A, salicylate-AMP ligase; NRPS, non-ribosomal peptide synthetase; Red, thiazoline reductase; P, permease (component of ABC transporter); ATP, ATPase (component of ABC transporter); ABC, ABC transporter; Re, putative oxidoreductase; α/β, putative α/β hydrolase]. See Table S2 for homology to other NRPS systems and transporters. B. Organization of modules and domains in the Eqb NRPS (ArCP, aryl acid carrier protein; Cy, heterocyclization; A, adenylation; E, epimerization; PCP, peptidyl carrier protein; MT, methyl transferase; Te, type I thioesterase). C. Proposed intermediates in equibactin biosynthesis.

The ‘cargo’ region between the conjugation and recombination modules that encodes vancomycin resistance in Tn*1549* carries transport genes of unknown function in CDTn*2/5* and CDSs in ICE*Se2* with most overall similarity to the NRPS cluster 1 of *Clostridium kluyveri*, which is proposed to biosynthesize a putative siderophore (Seedorf *et al*., 2008). Several of the encoded proteins were also similar to the NRPS complex of *Yersinia* sp. that produces the ferric iron-binding siderophore yersiniabactin. Therefore, we hypothesized that the ICE*Se2* cargo region which contains the ‘*eqb*’ gene cluster is responsible for the biosynthesis of a novel thiazoline-containing non-ribosomal peptide that we have tentatively named ‘equibactin’ although it is possible that the product from this NRPS could be identical to previously identified molecules. The *eqb* cluster contains 14 coding sequences (*eqbA–N*), the putative functions of which include a DtxR-like repressor (*eqbA*), non-ribosomal peptide biosynthetic proteins (*eqbB–G*, *eqbMN*), a ferric-siderophore-like importer (*eqbH–J*) and ABC transporters (Fig. 1A).

The prevalence of the equibactin locus in diverse *S. equi* and *S. zooepidemicus* populations was determined by Southern blot with a probe specific to the *eqbN* gene and PCR of the *eqbE* gene using primers listed in Table S1. The *eqbN* and *eqbE* genes were detected in 18 of 18 *S. equi* isolates but 0 of 73 *S. zooepidemicus* isolates. In addition, the location of the ICE*Se2* as determined by Southern blot and PCR across flanking regions was identical in all 18 *S. equi* strains.

### Functions of the equibactin gene cluster as predicted by genetic analysis

Dissection of the modular structure revealed a remarkable similarity between the *C. kluyveri*, yersiniabactin and equibactin NRPS systems (Table S2). The first of the two peptide synthetases, EqbE, shares 45% and 30% aa sequence identity with CKL_1505 and HMWP2 respectively, while EqbG shares 25% aa sequence identity with CKL_1511 and HMWP1. Analagous to the characterized HMWP1 and HMWP2, EqbG and EqbE are comprised of four carrier domains, three cyclization domains, an epimerization domain, a single adenylation domain, a methyltransferase domain and a type I thioesterase domain (Fig. 1B). The EqbG protein has 1272 aa, but lacks the N-terminal 1895 aa of HMWP1 involved in polyketide synthesis (Miller *et al*., 2002). EqbC shares 23% aa identity with CKL_1523 (Table S2). Although lacking sequence homology, *eqbC* like *ybtD*, which is located outside of the *Yersinia pestis* HPI (Bobrov *et al*., 2002), encodes a putative 4′-phosphopantetheinyl transferase. EqbM shares 31% aa identity with CKL_1509 of *C. kluyveri* (Seedorf *et al*., 2008) and aligned to the Pfam Saccharopine dehydrogenase family, a member of the FAD/NAD(P)-binding Rossmann fold superfamily of redox enzymes. EqbN, a putative membrane protein, has a predicted N-terminal transmembrane helix and shares 31% aa identity with a hypothetical protein Csac_2163 of *Caldicellulosiruptor saccharolyticus*. EqbN and Csac_2163 aligned to the Pfam α/β hydrolase family of enzymes that contain a catalytic triad associated with diverse catalytic activity.

The three predicted ABC transporters encoded by *eqbHIJ*, *eqbK* and *eqbL* are homologous (34–54% aa sequence identity) to unknown ABC transporters in *Clostridium beijerinckii* (Cbei_3098, Cbei_3097, Cbei_3096, Cbei_3094 and Cbei_3093 respectively) (Table S2). EqbI, EqbJ, EqbK and EqbL also share 24–45% aa identity with the putative *C. kluyveri* proteins CKL_1516, CKL_1517, CKL_1512 and CKL_1513 respectively (Seedorf *et al*., 2008). EqbK and EqbL share 55–57% aa sequence identity with SAS2320 and SAS2319 in *Staphylococcus aureus* (Holden *et al*., 2004) and have a fused N-terminal permease and C-terminal ATPase. Normally this would suggest involvement in efflux of extracellular toxins (Saurin *et al*., 1999). However, EqbK and EqbL share 28–30% aa sequence identity with YbtP and YbtQ of *Y. pestis* which, like IrtA and IrtB of *Mycobacterium tuberculosis*, are involved in siderophore-mediated iron import and are structurally unique among the subfamily of ABC transporters associated with iron transport (Fetherston *et al*., 1999; Rodriguez and Smith, 2006).

### Substrate predictions

An important characteristic of the equibactin and yersiniabactin biosynthetic clusters is the presence of a single A-domain in HMWP2/EqbE that is probably responsible for the aminoacylation of all three different PCP domains (two in HMWP2/EqbE and one in HMWP1/EqbG), two of which are not linked physically to the A-domain. By comparison to the GrsA A_Phe_ prototype, critical residues lining the substrate-binding pocket of the EqbE A-domain active site were identified (Stachelhaus *et al*., 1999; Challis *et al*., 2000) and found to match those mediating cysteine recognition with the exception of a change from asparagine to aspartate at residue 278 (Table 1).

**Table 1:** Prediction of EqbD and EqbE A-domain substrate specificity

		Residue (according to A_Phe_ numbering)								
			
A-domain^a^	Substrate	235	236	239^b^	278	299	301	322	330^b^	Accession number
HMWP2	Cys	D	L	Y	N	M	S	M	I	Q9Z399
BacA	Cys	D	L	Y	N	L	S	L	I	O68006
AngR	Cys	D	L	Y	N	M	S	M	I	P19828
PchE	Cys	D	L	F	N	L	S	L	I	Q9RFM8
PchF	Cys	D	L	Y	N	L	S	L	I	Q9RFM7
CtaC	Cys	D	L	Y	N	M	S	L	V	Q5MD35
MtaC	Cys	D	L	Y	N	M	S	L	I	Q9RFK9
BlmIV	Cys	D	L	Y	N	L	S	L	I	Q9FB18
EqbE	Cys?	D	L	Y	**D**	M	S	M	I	
DhbE	DHB	N	Y	S	A	Q	G	V	V	P40871
EntE	DHB	N	Y	S	A	Q	G	V	V	P10378
MxcE	DHB	N	F	S	A	Q	G	V	V	Q9F638
VibE	DHB	N	F	S	A	Q	G	V	V	O07899
AngE	DHB	N	F	S	A	Q	G	V	V	Q5DK17
YbtE	Sal	N	F	C	A	Q	G	V	L	Q56950
PchD	Sal	N	F	C	A	Q	G	V	I	Q9RFM9
EqbD	Sal?	N	F	C	**G**	Q	G	I	I	
SnbA	3-Hydroxy-picolinic acid	N	F	C	S	Q	G	V	L	P95819

a. Protein name: HMWP2, yersiniabactin-NRPS, *Y. pestis* (Gehring *et al*., 1998b); BacA, bacitracin NRPS, *Bacillus licheniformis* (Konz *et al*., 1997); AngR, anguibactin NRPS, *Vibrio anguillarum* (Tolmasky *et al*., 1993); PchE and PchF, pyochelin NRPSs, *Pseudomonas aeruginosa* (Quadri *et al*., 1999); CtaC, cystothiazole A NRPS, *Cystobacter fuscus* (Feng *et al*., 2005); MtaC, myxothiazol NRPS, *Stigmatella aurantiaca* (Silakowski *et al*., 1999); BlmIV, bleomycin NRPS, *Streptomyces verticillus* (Du *et al*., 2000); DhbE, bacillibactin DHB-AMP ligase, *Bacillus subtilis* (May *et al.*, 2002); EntE, enterochelin synthase, *Escherichia coli* (Gehring *et al*., 1997); MxcE, myxochelin DHB-AMP ligase, *S. aurantiaca* (Silakowski *et al*., 2000); VibE, vibriobactin DHB-AMP ligase, *Vibrio cholerae* (Wyckoff *et al*., 1997); AngE, anguibactin DHB-AMP ligase, *V. anguillarum* (Alice *et al*., 2005); YbtE, yersiniabactin salicyl-AMP ligase, *Y. pestis* (Gehring *et al*., 1998b); PchD, pyochelin salicyl-AMP ligase, *P. aeruginosa* (Quadri *et al*., 1999); SnbA, pristinamycin I 3-hydroxy-picolinic acid-AMP ligase, *Streptomyces pristinaespiralis* (de Crecy-Lagard *et al*., 1997).

b. Amino acids at positions 239, 330 discriminate Sal from DHB (May *et al.*, 2002). *S. equi* residues defined in bold differ from the consensus code of characterized substrate-activating proteins.

The predicted amino acid sequences between core A4 and A5 sequence motifs of the EqbE A-domain and EqbD were aligned, using clustalw, to A-domains or aryl-AMP ligase homologues with > 30% sequence identity to EqbE or EqbD respectively (Stachelhaus *et al*., 1999). Based on the structural data of DhbE and GrsA, residues conferring substrate specificity were identified (Stachelhaus *et al*., 1999; May *et al.*, 2002).

EqbD shares 42% aa identity to the YbtE salicylate-AMP ligase (Gehring *et al*., 1998a), 41% sequence identity with 2,3-dihydroxybenzoate (DHB) AMP ligase, DhbE (May *et al.*, 2002), and 55% aa identity with the uncharacterized CKL_1504 of *C. kluyveri* (Seedorf *et al*., 2008). The adapted specificity conferring code for aryl acid-activating domains discriminates between DHB and salicylate-activating enzymes (May *et al.*, 2002). EqbD is predicted to activate salicylate since it has a cysteine at position 239, which would impede access of the 3′-OH group of DHB and a more sterically demanding isoleucine at position 330 that replaces the conserved valine in DHB-activating enzymes (Table 1). However, a small change from alanine to glycine was identified relative to residue 278 according to GrsA A_phe_ numbering, which differs from other NRPS systems that utilize salicylate.

### Proposed mechanism of equibactin biosynthesis

**Fig. 2:**
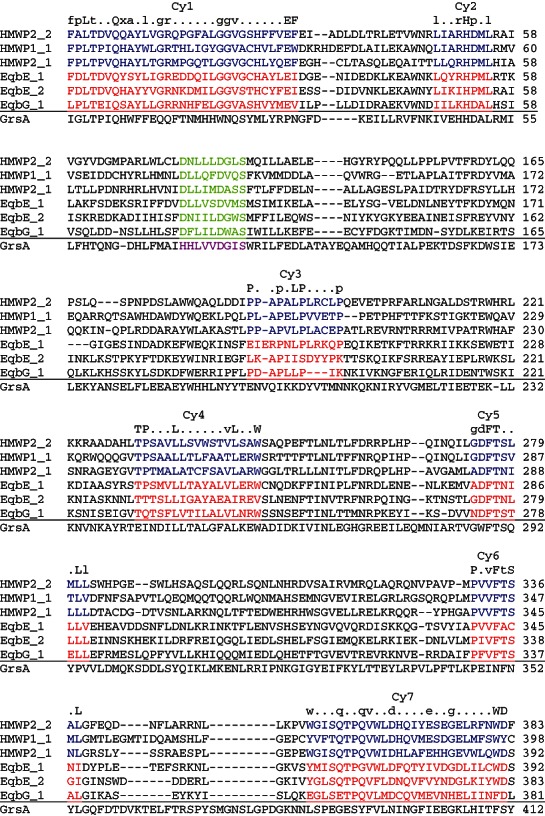
Bioinformatic prediction of *eqb* NRPS substrates. clustalw alignment of amino acid sequences of the putative Cy domains of EqbE, EqbG *S. equi*, HMWP1, HMWP2 *Y. pestis* and the classical condensation domain of GrsA *Bacillus brevis* (below line). Highlighted areas (red: *S. equi*, blue: *Y. pestis*) represent the seven conserved signature sequences (Cy1–Cy7) with the consensus shown above (Konz *et al*., 1997). The DX_4_DX_2_S catalytic core containing the two aspartate residues critical to amide bond formation and heterocyclization (Keating *et al*., 2000) is shown in green. The HHX_3_DGXS catalytic core of classic condensation domains is shown in purple for GrsA (Konz *et al*., 1997).

Three condensation (C) domains identified in the two peptide synthetases, EqbE and EqbG, are strongly modified, diverging from classical C-domains involved in peptide bond formation. Instead, C-domains shared the highest homology (29–32% aa sequence identity) with cyclization (Cy) domains that catalyse thiazoline ring formation in yersiniabactin and bacitracin biosynthesis (De Crecy-Lagard *et al*., 1995; Konz *et al*., 1997; Bobrov *et al*., 2002). Seven signature regions identified in Cy domains of *Yersinia enterocolitica* (yersiniabactin), *Bacillus licheniformis* (bacitracin) and *Vibrio anguillarum* (anguibactin) by Konz *et al*. (1997) were also mostly conserved in the three putative Cy domains of EqbE and EqbG (Fig. 2). Some differences in these sequence alignments were apparent although the contribution of residues in these regions to catalytic activity is not yet known. However, the highly conserved Cy motif D-x-x-x-x-D-x-x-S that corresponds in location to the highly conserved condensation domain catalytic core, H-H-x-x-x-D-G-x-S (Konz *et al*., 1997; Keating *et al*., 2000), is present in all three putative Cy domains of *S. equi.* This suggests an involvement of these putative Cy domains in heterocylization activity since the two asparate residues of this Cy core are essential to both amide bond formation and heterocyclization by Cy1 of HMWP2 in *Y. pestis* (Keating *et al*., 2000).

The presence of a putative thiazoline reductase (EqbF) with 32% aa identity with YbtU (Gehring *et al*., 1998a) and 32% and 31% aa identity with CKL_1506 and CKL_1510 of *C. kluyveri*, respectively (Seedorf *et al*., 2008), supports a prediction of thiazoline rings in the chemical structure of equibactin. Thiazoline rings of yersiniabactin and pyochelin are reduced to thiazolidine by the activity of these thiazoline reductase enzymes (Patel and Walsh, 2001; Miller *et al*., 2002). These enzymes also have a methyl transferase module, which, although apparently non-functional in yersiniabactin biosynthesis, introduce a methyl group to the N-atom of the thiazolidine residue in pyochelin (Patel and Walsh, 2001; Miller *et al*., 2002). A prediction of the NRPS intermediates is presented in Fig. 1C.

Non-ribosomal peptides are usually released from the last carrier domain in the assembly line through the activity of a terminal thioesterase (type I) (Challis and Naismith, 2004). The C-terminal domain of EqbG has similarity with type I thioesterases. However, sequence alignment of this domain to thioesterases in Pfam showed a S1092 to A mutation in the critical serine residue (GXSXG motif) of the predicted serine, histidine, aspartate catalytic triad essential for function (Bobrov *et al*., 2002; Reimmann *et al*., 2004). Alignment of the type II thioesterase encoded by *eqbB* showed an intact catalytic triad.

### Regulation of the *eqb* NRPS

Upstream of the *eqb* operon is a CDS predicted to encode a putative metal ion-dependent repressor, EqbA. Close homologues of *eqbA* and *eqbD* gene products are present in the genome of *S. agalactiae* serotype III NEM316, although the latter gene appears to be a pseudogene in this group B streptococcus. EqbA is predicted to have the characteristic N-terminal DNA binding, central metal ion binding and dimerization domains of the MntR and IdeR family of manganese and iron-dependent repressors to which it has 33% and 28% sequence identity respectively (Schmitt and Holmes, 1991; Que and Helmann, 2000). DtxR, a member of the growing family of IdeR global iron-dependent regulators in Gram-positive bacteria, is activated on binding of divalent iron. Subsequent binding of the homodimeric form to a 21 bp DNA duplex blocks the transcription of downstream genes (Schmitt and Holmes, 1991; Pohl *et al*., 1999a). A unique 36 bp imperfect palindrome (underlined) was identified in the promoter region of *eqbB* and represents a potential operator sequence for EqbA:

5′-AACTATTATTGTTAGATGTAT**CTAACA**ATAATAGTTCTAGTAG**TATATT**AATAATCAGATGGAAGGTGTTTTG**ATG**-3′ (−35, −10 promoter sites and the ATG translational start are highlighted in bold).

**Fig. 3:**
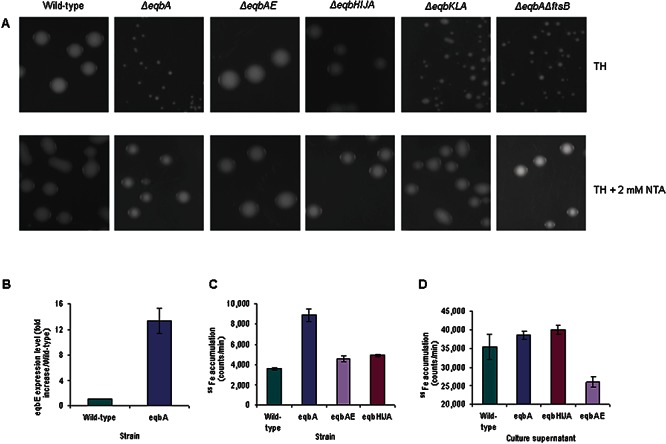
Phenotypic effects of deletions in the *eqb* gene cluster. A. Photographs show colonies of wild-type, Δ*eqbA*, Δ*eqbAE*, Δ*eqbHIJA*, Δ*eqbKLA* and Δ*eqbA*Δ*ftsB S. equi* strains grown overnight on THA and the increase in colony size of the Δ*eqbA*, Δ*eqbKLA* and Δ*eqbA*Δ*ftsB* strains grown on THA supplemented with 2 mM NTA. B. Fold increase in *eqbE* transcript level in the Δ*eqbA* strain relative to the wild-type 4047 strain, which has been normalized to one (mean ± standard error mean, *n* = 3). C. Quantification of ^55^Fe accumulation by different *S. equi* strains (mean ± standard error mean, *n* = 3). The difference in ^55^Fe accumulation between the Δ*eqbA* strain and the Δ*eqbAE*, Δ*eqbHIJA* and wild-type strains was found to be statistically significant using two-sample Wilcoxon rank-sum (Mann–Whitney) tests (*P* = 0.05, *n* = 3). D. Quantification of ^55^Fe accumulation by *S. equi* strain Δ*eqbAE* cross-fed with filter-sterilized culture supernatant from different *S. equi* strains grown to stationary phase.

To determine the role of EqbA on the regulation of the *eqb* NRPS, we generated a series of allelic replacement mutants in *S. equi* strain 4047. Deletion of *eqbE* had no effect on the growth rate of *S. equi* on Todd–Hewitt agar (THA). However, deletion of *eqbA* produced very small colonies (Fig. 3A). We hypothesized that this phenotype resulted from iron toxicity resulting from over-production of the product(s) of the *eqb* cluster. Over-expression of *eqbE* was confirmed since deletion of *eqbA* resulted in a 13-fold increase in *eqbE* transcript levels (Fig. 3B). The generation of an Δ*eqbA*, Δ*eqbE* double deletion strain (Δ*eqbAE*), which had a large colony phenotype on THA, established that the slow growth of the Δ*eqbA* strain was as a direct consequence of the function of the *eqbE* gene product (Fig. 3A). Transformation of the Δ*eqbA* or Δ*eqbAE* strains with the pGhost9 plasmid containing a second copy of *eqbA* under the control of the *eqbA* promoter or a second copy of *eqbE* under the control of the *eqbB* promoter complemented the Δ*eqbA* and Δ*eqbE* and induced a large or small colony phenotype respectively (Fig. S2). The lack of an effect on the colony phenotype of *S. equi* grown *in vitro* following deletion of *eqbE* may be due to continued import of cations through the activity of several alternative cation transport systems, which include an HtsABC haem-binding system (SEQ0445 to SEQ0448) (Nygaard *et al*., 2006), a putative MtsABC Mn^2+^ and Fe^3+^ metal transport system (SEQ1658 to SEQ1660) with 80–91% aa sequence identity to that of *S. pyogenes* (Janulczyk *et al*., 1999) and a putative FtsABCD Fe^3+^ ferrichrome transport system (SEQ1836 to SEQ1839) with 59–77% aa sequence identity to that of *S. pyogenes* (Hanks *et al*., 2005) (http://www.sanger.ac.uk).

Further studies supported the hypothesis that the *eqbA* deletion phenotype resulted from increased iron uptake. Supplementation of THA with 2 mM nitrilotriacetic acid (NTA), a known chelator of iron, restored near normal colony size in the Δ*eqbA* strain (Fig. 3A). Susceptibility to streptonigrin was used as an indirect measure of intracellular iron concentration (Brown *et al*., 2002). Growth of wild-type *S. equi* was prevented by a minimal inhibitory concentration of 0.06 μM streptonigrin in THB. The Δ*eqbA* mutant was 16 times more sensitive to streptonigrin, while strains predicted to be unable to produce the NRPS product(s) through deletion of the biosynthetic gene *eqbE* (Δ*eqbE* and Δ*eqbAE*) were twice more resistant to the antibiotic (Table 2A). There was no difference in susceptibility of these strains to the antibiotic erythromycin. Therefore, increased sensitivity of the Δ*eqbA* mutant to streptonigrin compared with wild-type, Δ*eqbE* or Δ*eqbAE* strains suggests a greater intracellular iron pool in the former most likely as a result of de-repression of *eqb* non-ribosomal peptide synthesis. The same differences in streptonigrin sensitivity were conferred when the Δ*eqbAE* mutant was cross-fed with filter-sterilized stationary-phase culture supernatant from the wild-type and various mutant strains (Table 2B), consistent with an accumulation of iron being mediated by an excreted, soluble factor.

**Table 2:** Production of the *eqb* NRPS product by allelic replacement mutants of *S. equi* and *E. coli* strains expressing different complements of the Eqb proteins

A		

Strain	Streptonigrin MIC(μM)	Erythromycin MIC (μg ml^−1^)
Wild type	0.06	0.016
Δ*eqbA*	0.004	0.016
Δ*eqbE*	0.125	0.016
Δ*eqbHIJ*	0.06	0.016
Δ*eqbKL*	0.06	0.016
Δ*eqbAE*	0.125	0.016
Δ*eqbHIJA*	0.03	0.016

B		

Conditioned THB from strain	Streptonigrin MIC of strain Δ*eqbAE* (μM)	Erythromycin MIC of strain Δ*eqbAE* (μg ml^−1^)
Wild type	0.03	0.016
Δ*eqbA*	0.004	0.016
Δ*eqbE*	0.125	0.016
Δ*eqbAE*	0.125	0.016
Δ*eqbHIJ*	0.016	0.016
Δ*eqbKL*	0.06	0.016
Δ*eqbHIJA*	0.002	0.016

C			

Conditioned CDM from strain	Supplementation	Streptonigrin MIC of strain Δ*eqbAE* (μM)	Erythromycin MIC of strain Δ*eqbAE* (μg ml^−1^)
Δ*eqbAE*		0.125	0.03
Δ*eqbHIJA*		0.125	0.03
Δ*eqbAE*	10 μM salicylate	0.125	0.03
Δ*eqbHIJA*	10 μM salicylate	0.0005	0.03

D			

Conditioned media from *E. coli*expressing Eqb proteins	Media	Streptonigrin MIC of strain Δ*eqbAE* (μM)	Erythromycin MIC of strain Δ*eqbAE* (μg ml^−1^)
EqbBCDEFGMN	LB	0.06	0.004
EqbBCDEFGMN	LB + 1 mM salicylate	0.002	0.004
EqbBCDFGMN + pET21a (no EqbE)	LB + 1 mM salicylate	1	0.004
EqbBCDEFMN (no EqbG)	LB + 1 mM salicylate	1	0.004
EqbBCDEFGN (no EqbM)	LB + 1 mM salicylate	1	0.004
EqbBCDEFGM (no EqbN)	LB + 1 mM salicylate	0.5	0.004
EqbBCDEFGMN	MM + 1 mM salicylate	0.002	0.016
EqbBCDFGMN + pET21a (no EqbE)	MM + 1 mM salicylate	0.06	0.016

A. Sensitivity of wild-type and allelic replacement strains of *S. equi* to streptonigrin and erythromycin. MIC refers to the minimum inhibitory concentration of antibiotic required to prevent growth. The twofold difference in the streptonigrin MIC of wild type versus Δ*eqbE* was found to be statistically significant using a two-sample Wilcoxon rank-sum (Mann–Whitney) test (*P* = 0.008, *n* = 4).

B. Streptonigrin and erythromycin sensitivity in the Δ*eqbAE* strain cross-fed with filter-sterilized culture supernatant from wild-type and allelic replacement strains grown to stationary phase in THB. The twofold difference in Δ*eqbAE* streptonigrin MIC conferred by cross-feeding with Δ*eqbA*-conditioned CDM relative to Δ*eqbHIJA*-conditioned CDM was found to be statistically significant using a two-sample Wilcoxon rank-sum (Mann–Whitney) test (*P* = 0.008, *n* = 4).

C. Streptonigrin and erythromycin sensitivity in the Δ*eqbAE* strain cross-fed with filter-sterilized culture supernatant from Δ*eqbAE* and Δ*eqbHIJA* allelic replacement strains grown to stationary phase in CDM ± 10 μM salicylate.

D. Streptonigrin and erythromycin sensitivity in the Δ*eqbAE* strain cross-fed with filter-sterilized culture supernatant from *E. coli* strains expressing different complements of Eqb proteins grown to stationary phase in LB ± 1 mM salicylate or MM + 1 mM salicylate.

The increased production of equibactin in the Δ*eqbA* mutant was further supported by ^55^FeCl_3_ incorporation assays, which showed an almost twofold increase in intracellular iron in the repressor deletion strain, compared with wild type or the Δ*eqbAE* mutant (Fig. 3C). Δ*eqbAE* cross-fed with filter-sterilized stationary-phase culture supernatant from the Δ*eqbA* strain but not the Δ*eqbAE* strain had a similar increase in ^55^FeCl_3_ accumulation (Fig. 3D).

### The mechanism for import of the NRPS product(s)

We exploited the small colony phenotype of the Δ*eqbA* mutant to identify other CDSs likely to be involved in *eqb* product function. Deletion of the putative ABC transporters encoded by *eqbK* and *eqbL* or the *ftsB* gene, which lies outside the *eqb* locus and encodes a putative ferric-siderophore receptor (Hanks *et al*., 2005; Clancy *et al*., 2006), did not prevent the generation of the small colony phenotype on subsequent deletion of *eqbA* (Fig. 3A). However, deletion of the *eqbH*, *eqbI* and *eqbJ* genes, followed by deletion of *eqbA* (Δ*eqbHIJA*), did prevent the generation of the small colony phenotype (Fig. 3A). Reduced levels of ^55^Fe in the Δ*eqbHIJA* strain also suggested that the *eqbH*, *eqbI* and/or *eqbJ* gene products are essential for the majority of *eqb* product-dependent iron accumulation (Fig. 3C).

To determine if *eqbH*, *eqbI* and *eqbJ* were important to the export or import of the NRPS product(s), filter-sterilized culture supernatant from mutant strains was added to the Δ*eqbAE* strain and its susceptibility to streptonigrin was quantified. Media from the Δ*eqbHIJA* strain increased the sensitivity of the Δ*eqbAE* strain by 64-fold compared with a 32-fold increased sensitivity conferred by media from the Δ*eqbA* strain (*P* = 0.008, Table 2B). These data suggest that the Δ*eqbHIJA* strain secretes, but is unable to import the NRPS product(s) resulting in a build-up of this product in the culture media. In addition, Δ*eqbAE* cross-fed with filter-sterilized culture supernatant from the Δ*eqbHIJA* strain had an increase in ^55^FeCl_3_ accumulation slightly greater, although not significantly so, than that observed when Δ*eqbAE* was cross-fed with supernatant from the Δ*eqbA* strain (Fig. 3D).

### The biochemical requirements for the *eqb* NRPS

A homologue of the salicylate synthases encoded by *ybtS* in *Yersinia* sp. (Miller *et al*., 2002) or *pchA* and *pchB* of *Pseudomonas* sp. (Serino *et al*., 1995) was not identified in *S. equi* strain 4047. *S. equi* cultured in Todd–Hewitt medium prepared from bovine heart infusion and a tryptic digest of animal tissue required no supplementation for equibactin production. However, production of the *eqb* product by the Δ*eqbHIJA* strain in chemically defined media (CDM) required salicylate supplementation consistent with salicylate being a substrate for the equibactin NRPS (Table 2C). The CDM contains 0.2 mM l-cysteine.

### Electrophoretic mobility shift assay

**Fig. 4:**
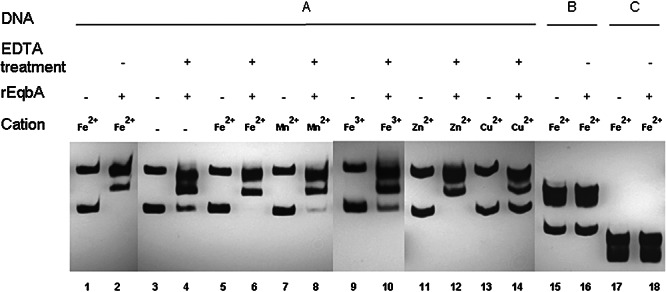
Electrophoretic mobility shift assay. Biotin end-labelled DNA fragments containing upstream regions of *eqbB*, A (227 bp, −237 to −11 bp), B (165 bp, −237 to −73 bp) or C (103 bp, −237 to −135 bp), were incubated in the presence of Fe^2+^, Mn^2+^, Fe^3+^ Zn^2+^, Cu^2+^ or control (−) as indicated with (+) or without (−) rEqbA and with (+) or without (−) prior treatment of rEqbA with EDTA. DNA fragments B and C lack the palindromic promoter region (−73 to −38 bp) immediately upstream of *eqbB*. Experimental conditions are described in *Experimental procedures*.

Electrophoretic mobility shift assays were used to analyse recombinant EqbA (rEqbA) binding to a 227 bp DNA fragment (A) containing the upstream region of the *eqb* operon (P_eqb_) (−237 to −11 bp) (Fig. 4). Unbound DNA ran as two distinct bands on non-denaturing polyacrylamide gels and was unaffected by the presence of different cations (lanes 1, 3, 5, 7, 9, 11 and 13). Binding of rEqbA caused a shift in the mobility of the lower band (lane 2). Pre-treatment of rEqbA with EDTA resulted in a partial shift in the mobility of the lower band (lane 4), which could be enhanced in the presence of additional Fe^2+^ or Zn^2+^ (lanes 6 and 12) and to a lesser degree by Mn^2+^ (lane 8), but not by Fe^3+^ or Cu^2+^ (lanes 10 and 14). The presence of the −73 bp to −38 bp DNA palindrome was essential for rEqbA binding as no shift was observed using DNA fragments containing the −237 to −73 bp (B) (lanes 15 and 16) or −237 to −135 bp (C) (lanes 17 and 18) upstream regions of the *eqb* operon. Incubation of a control DNA fragment [LightShift® Chemiluminescent EMSA Kit (Pierce)] with rEqbA did not result in a shift in mobility and a control crude extract [LightShift® Chemiluminescent EMSA Kit (Pierce)] did not bind to P_eqb_ (data not shown), suggesting that rEqbA binds specifically to P_eqb_.

### Reconstitution of the equibactin NRPS in *E. coli*

To determine if all of the genes necessary for production of equibactin were present in the *eqb* locus, and to produce equibactin in a less complex media, we cloned *eqbB*, *eqbC*, *eqbD*, *eqbE*, *eqbF*, *eqbG*, *eqbM* and *eqbN* into three expression plasmids with compatible replicons. Production of all eight Eqb proteins was induced by IPTG treatment of transformed *E. coli* strain BL21(DE3) cells grown to log phase in LB broth or a minimal medium (MM) in the presence of 1 mM salicylate as in *Experimental procedures*.

Filter-sterilized supernatant from *E. coli* containing all eight *eqb* CDSs and grown in LB increased the sensitivity of strain Δ*eqbAE* to streptonigrin from 1 μM to 0.002 μM, but had no effect on the sensitivity to erythromycin in cross-feeding assays (Table 2D). Exclusion of *eqbE*, *eqbG*, *eqbM* or *eqbN* prevented the associated increased streptonigrin sensitivity suggesting that all four of these proteins are required for reconstitution of the equibactin NRPS in *E. coli* (Table 2D).

An increased sensitivity to streptonigrin on cross-feeding strain Δ*eqbAE* with filter-sterilized MM following growth of *E. coli* containing all eight *eqb* CDSs from 0.06 to 0.002 was also observed (Table 2D).

However, despite these effects on the streptonigrin sensitivity of strain Δ*eqbAE* in cross-feeding assays, repeated LC-MS analysis of culture supernatants, including conditioned MM, failed to identify equibactin.

## Discussion

Available evidence suggests that *S. equi* evolved from an ancestral *S. zooepidemicus* strain (Jorm *et al*., 1994; Webb, 2008), an opportunistic zoonotic pathogen largely associated with subclinical inflammatory airway disease in young horses (Wood *et al*., 2005). Given the close genetic similarity between these two streptococci, we used comparative genomics to identify differences between the species with a view to distinguishing key determinants of their differing pathogenic properties. The full results of our genome comparison will be reported elsewhere. However, in addition to previously described genetic differences (Galan and Timoney, 1987; Timoney *et al*., 1997; Lindmark *et al*., 1999; Artiushin *et al*., 2002; Proft *et al*., 2003; Alber *et al*., 2005) we report here the identification of a streptococcal ICE, *ICESe2*, and the function of the siderophore-like NRPS system encoded by this element. This element was previously noted in a broad screen for ICEs in sequenced bacterial genomes, but no putative function was assigned (Burrus *et al*., 2002b).

ICE*Se2* was present in all strains of *S. equi*, but absent from all strains of *S. zooepidemicus* examined. These strains were selected to be a diverse population based on their SeM allele (Kelly *et al*., 2006; Waller and Jolley, 2007) or 16S−23S RNA gene intergenic spacer/SzP PCR subtype (Newton *et al*., 2008) and were highly diverse based on their MLST sequence type (Webb, 2008). Our results also indicated that ICE*Se2* was located at the same genome position in all 18 *S. equi* strains, suggesting that its integration was an early and stable event in the evolution of *S. equi*.

The similarity of ICE*Se2* CDSs predicted to be involved in conjugation and site-specific recombination to those from *C. difficile* CDTn*2* and CDTn*5* is extremely interesting and raises the possibility of acquisition of the equibactin NRPS locus via a conjugative-like mechanism from a genetically unrelated bacterium. Although we predict integrative and conjugative genes are present in ICE*Se2*, to date we failed to detect a circular intermediate by PCR through repeated experiments suggesting that this element may no longer retain the ability to recircularize and transfer to other bacteria.

ICE*Se2*, CDTn*2* and CDTn*5* have putative conjugation modules that are closely related to the conjugation module of Tn*1549* in *Enterococcus* spp., suggesting that they have inherited their similar structure from a common module ancestor. However, the serine recombinase encoded at the right flank of these elements differs from the Int (tyrosine recombinase) and Xis of Tn*1549*, which taken together with the differences in accessory functions highlights the importance of module exchange in the evolution of novel mosaic ICE structures. Serine recombinases are less common and genetically unrelated to the tyrosine recombinases and use a different mechanism for the site-specific recombination of mobile elements (Grindley *et al*., 2006). However, this family is rather heterogeneous and ranges in size from 180 to nearly 800 aa residues. ICE*Se2* encodes a small recombinase (SEQ1229) with homology to the N-terminal half of the large TndX resolvase, which mediates excision and insertion of Tn*5397* by introducing 2 bp staggered cuts at the 3′ ends of GA dinucleotides at the ends of the element (excision) or in the target site (integration) (Wang *et al*., 2000). Consequently, direct GA dinucleotide repeats delineate the ends of Tn*5397* and one copy is also present at the joint of the circular form. CDTn*5* also has 5 bp direct repeats at these corresponding sites, whereas no direct repeats were identified in the flanks of ICE*Se2*. However, particularly large 108 bp perfect inverted repeats were present at the flanks of the element in *S. equi*, which like the much smaller imperfect inverted repeats (19–20 bp) in Tn*5397* and other ICEs may represent DNA binding sequences for the recombinase during the formation of the synaptic complex.

Reconstitution of the equibactin NRPS in *E. coli* was successfully achieved using three compatible expression plasmids containing *eqbB*, *eqbC*, *eqbD*, *eqbE*, *eqbF*, *eqbG*, *eqbM* and *eqbN*. Production in *E. coli* was enhanced by supplementation of growth media with 1 mM salicylate, was abolished in the absence of *eqbE*, *eqbG* and *eqbM* and was reduced in the absence of *eqbN*. Therefore, each of these genes plays a role in the biosynthesis of the *eqb* NRPS product(s) and the equibactin locus contains all of the genes unique to *S. equi* that are required for the biosynthesis of NRPS product(s).

The sequence of a NRPS has been used previously to predict the structure of the peptide produced (Challis and Ravel, 2000; Lautru *et al*., 2005). The similarity in type and organization of functional modules in the equibactin NRPS and the yersiniabactin biosynthetic system (Miller *et al*., 2002) leads us to suggest a tentative model for equibactin biosynthesis (Fig. 1). Initially, we propose activation of the aryl acid carrier protein (ArCP) domain of EqbE and the peptide carrier domains (PCP) of EqbE and EqbG by the putative 4′-phosphopantetheinyl transferase encoded by *eqbC*. EqbD is proposed to activate salicylate, which is then transferred to the phosphopantotheine thiol of the ArCP domain of EqbE. Our observation that addition of salicylate is required for production of equibactin in CDM provides evidence in support of the proposed involvement of salicylate. Salicylate was also required for optimum production of equibactin in *E. coli* containing the reconstituted NRPS, but was not required for production of equibactin by *S. equi* in Todd–Hewitt medium. This medium is prepared from bovine heart infusion and a tryptic digest of animal tissue and we propose that an appropriate NRPS substrate is released from host tissue during infection with *S. equi*. The single adenylation (A)-domain of EqbE is predicted to activate cysteine which is then transferred to the phosphopantotheine thiol of each of the three PCP domains of the NRPS (two in EqbE and one in EqbG). We predict that the cysteinyl thioesters attached to each PCP domain will be condensed and cyclized as the growing chain translocates to each of the three PCP domains. The presence of a putative thiazoline reductase (EqbF) suggests that as in yersiniabactin biosynthesis, the second thiazoline ring of equibactin is likely to be reduced to a thiazolidine to prevent the auto-oxidation of the first ring to a thiazole. However, the available data do not rule out the possibility that the first or third thiazoline ring could be reduced to a thiazolidine instead of or as well.

Yersiniabactin is released from the last PCP domain by a C-terminal thioesterase domain. However, the C-terminal thioesterase domain of EqbG has the critical serine residue (S1092) of the predicted serine, histidine, aspartate catalytic triad essential for function (GXSXG motif) mutated to alanine. The type II thioesterase encoded by *eqbB* has an intact catalytic triad but to date these type II TEs have only a presumed editing role in maintaining the efficiency of non-ribosomal peptide synthesis via removal of inappropriate substrates (Marahiel *et al*., 1997; Schneider and Marahiel, 1998; Butler *et al*., 1999; Bobrov *et al*., 2002; Reimmann *et al*., 2004). Interestingly, the tetrapeptide coelichelin is synthesized by a trimodular NRPS lacking a TE domain and is proposed to be released through the action of a separately encoded hydrolase (Lautru *et al*., 2005). EqbN encodes a putative α/β hydrolase which could provide another potential mechanism for hydrolytic chain release similar to coelichelin. The *eqbN* gene was required for optimal production of the *eqb* NRPS product(s) by *E. coli* and induction of increased streptonigrin sensitivity in cross-feeding assays. However, absence of *eqbN* did not abolish production of the NRPS product(s) in this system suggesting that *E. coli* may produce an endogenous hydrolase that can complement the activity of EqbN or that direct hydrolysis with water without the formation of an acyl enzyme intermediate is also possible, either at a low background level or through a base-catalysed attack of water rather than nucleophilic attack of an active site serine (Li and Bugg, 2007).

Regulation of *eqb* non-ribosomal peptide synthesis is achieved through the action of the IdeR-like transcriptional repressor EqbA. Deletion of *eqbA* led to increased transcription of the NRPS operon, over-production of the NRPS product(s) and increased import of iron causing toxicity and a small-colony phenotype. Deletions of *ideR* and other *ideR/fur* homologues have been lethal in *M. tuberculosis* and several species of *Pseudomonas*, *Vibrio* and *Neisseria* (Berish *et al*., 1993; Tolmasky *et al*., 1994; Venturi *et al*., 1995). In *M. tuberculosis*, a rare deletion of *ideR* was obtained when the lethal effects of *ideR* inactivation were alleviated by a second-site suppressor mutation which restricted iron assimilation capacity (Rodriguez *et al*., 2002). We propose that like other IdeR family members, EqbA is activated on binding iron, leading to homodimerization and binding to a 36 bp DNA palindrome located immediately upstream of the NRPS operon to block its transcription. EqbA has a number of novel features compared with other IdeR family members. The suggested operator site is unlike other palindromic DNA iron boxes and this is reflected in the substitution of N-terminal helix–turn–helix residues involved in DNA contact (Fig. S3). Some of the conserved metal binding and dimerization residues differ in EqbA. In addition, the SH_3_-like fold of DtxR and IdeR, which functions to stabilize the DNA binding conformation of these repressors (Pohl *et al*., 1999b; Oram *et al*., 2005), is lacking in EqbA. EqbA is therefore likely to represent a new subclass of the IdeR family. Electrophoretic mobility shift assays confirmed that EqbA does indeed bind to the *eqbB* promoter in a Fe^2+^-, Zn^2+^- and Mn^2+^-responsive manner. EqbA binding was not affected by Cu^2+^ or Fe^3+^ and was dependent on the presence of the *eqbB* promoter's DNA palindrome. The shift due to addition of Mn^2+^ was not complete and may reflect a reduced affinity of EqbA for Mn^2+^. These data are in broad agreement with the activation of IdeR by different cations rather than MntR, which is Mn^2+^ specific (Schmitt *et al*., 1995; Que and Helmann, 2000; Guedon and Helmann, 2003; Chou *et al*., 2004). Further comparison of the putative residues involved in the co-ordination of the regulatory metal (Guedon and Helmann, 2003) revealed that EqbA shares a cysteine at aa position 104 with DtxR, which confers Fe^2+^ responsiveness rather than the Mn^2+^-responsive glutamate possessed by MntR and TroR at the homologous position (Fig. S3). The Fe^2+^-responsive methionine of DtxR has been replaced by asparagine at aa position 13 of EqbA, which is similar to the homologous residue in the Mn^2+^-responsive TroR.

No iron-chelating activity could be detected in the Δ*eqbHIJA* mutant of *S. equi* using the universal chrome azurol S (CAS) assay for iron chelators (Schwyn and Neilands, 1987). We have also been unable to identify equibactin by comparative metabolic profiling of the Δ*eqbA*, Δ*eqbAE* and Δ*eqbHIJA* strains using LC-MS. Furthermore, examination of biologically active conditioned LB and MM from *E. coli* containing the reconstituted equibactin NRPS by LC-MS also failed to identify equibactin. These data suggest that the concentration of equibactin in culture supernatant is low or the product of the *eqb* cluster may have a low affinity for iron and a structure different from that proposed. Pyochelin, a siderophore that lacks the third incorporated thiazoline and malonyl linker of yersiniabactin, but otherwise shares the salicyl-bis-thiazonyl core has a relatively low affinity for iron. A role for pyochelin in the uptake of other essential metals has been proposed (Visca *et al*., 1992) and this may also represent an alternative function for the *eqb* NRPS product(s). The host catecholamine norepinephrine, although unable to remove iron from CAS, can increase the availability of free iron to bacteria through an interaction that interferes with host glycoprotein iron sequestration (Freestone *et al*., 2000).

These data are also consistent with a signalling role for the NRPS product(s) in the upregulation of iron transport system(s). A number of siderophores are able to function as signalling molecules, influencing the expression of their own biosynthetic genes and cell surface receptors as well as other secreted virulence factors (Crosa, 1997; Pelludat *et al*., 1998; Beare *et al*., 2003; Michel *et al*., 2005; 2007; Miethke and Marahiel, 2007). Ferric-pyochelin acts as an intracellular effector via a direct interaction with an AraC-type regulator (Michel *et al*., 2005). The AraC-type YbtA regulator also induces yersiniabactin operons involved in yersiniabactin biosynthesis, Fe-yersiniabactin uptake and salicylate synthesis, and the Fe-yersiniabactin receptor. An interaction of the YbtA with its DNA promoter target and Fe-yersiniabactin is proposed to be a prerequisite for the recruitment of the RNA polymerase complex (Fetherston *et al*., 1996; Perry *et al*., 1999; Anisimov *et al*., 2005a, b; Miethke and Marahiel, 2007).

Siderophore-mediated iron uptake operons, similar to the ferric hydroxamate uptake (*fhu*) systems of *S. aureus* and *Bacillus subtilis*, have been identified in other streptococci (Hanks *et al*., 2005; Clancy *et al*., 2006; Pramanik and Braun, 2006). FhuD, the associated lipoprotein receptor in *S. agalactiae*, is able to bind a range of siderophores of both hydroxamate and catecholate classes. The homologous operon (42–55% aa sequence identity) is also present in the *S. equi* genome and we hypothesized that this could be involved in acquisition of the *eqb* NRPS product(s). However, deletion of the *ftsB* gene did not prevent iron toxicity due to over-production of the NRPS product(s) on the further deletion of *eqbA* (Fig. 3A). This indicates that FtsB is not absolutely required for import of the NRPS product(s) and further studies are necessary in order to determine if surface receptors play a role in import of the NRPS product(s). EqbK and EqbL share 29% aa sequence identity with YbtP and YbtQ of *Y. pestis* which, like IrtA and IrtB of *M. tuberculosis*, are involved in siderophore-mediated iron import and are structurally unique among the subfamily of ABC transporters associated with iron transport (Fetherston *et al*., 1999; Rodriguez and Smith, 2006). Surprisingly, deletion of *eqbK* and *eqbL* did not prevent the generation of a small colony phenotype on deletion of *eqbA* (Fig. 3A). Similar fused permease-ATPase type proteins have been associated with the efflux of substrates and the small-colony phenotype observed for *eqbKLA* triple mutant could result from a failure to secrete the siderophore (Saurin *et al*., 1999). Supernatant cross-feeding studies have consistently demonstrated that strain Δ*eqbKL* confers twofold decreased sensitivity to streptonigrin relative to wild type (Table 2B) suggesting that *eqbK* and/or *eqbL* could be involved in efflux of the NRPS product(s). The ABC transporter encoded by the *eqbH*, *eqbI* and *eqbJ* genes plays a major role in the *eqb*-dependent incorporation of iron. Purification and elucidation of the *eqb* NRPS product structure is warranted to better understand the role played by the NRPS product(s) *in vivo.*

Siderophore biosynthesis has not been identified in any streptococci examined to date (Eichenbaum *et al*., 1996). A non-ribosomal peptide synthesis system has been noted in the published genome sequence of the oral pathogen *S. mutans*. Unlike the *eqb* locus, this NRPS appears to incorporate five diverse residues into a molecule that is predicted to have more of an antibiotic-like structure (gramicidin/bacitracin family). However, homologues of *eqbA* and *eqbD* (pseudogene) are present in the genome of *S. agalactiae* serotype III, suggesting that a locus with similarity to the *eqb* NRPS may have been important to this organism at some point in its history.

ICE*Se2* is a key feature in the evolved genome of *S. equi*. While we have not yet identified the peptide made by the equibactin NRPS or shown direct iron binding, data consistent with its role in iron acquisition have been established. Given the importance of iron acquisition to other streptococcal pathogens (Brown and Holden, 2002), the acquisition of ICE*Se2* may have contributed to the increased pathogenesis of this important streptococcus. We hypothesize that more efficient acquisition of iron enhances retropharyngeal lymph node abscessation, which is critical to the establishment of long-term carriage and vital to the success of *S. equi*. Further studies to quantify the *in vivo* effects of the equibactin NRPS are now required to better understand its pathological significance.

## Experimental procedures

### Bacterial strains, media and growth conditions

*Streptococcus equi* strain 4047 was isolated in 1990 from the submandibular abscess of a New Forest pony. Strains were cultured in Todd–Hewitt broth (THB) (Sigma) or on THA (Sigma) at 37°C with 5% CO_2_ unless otherwise stated. CDM for Group A Streptococci (SAFC Biosciences) was used to produce conditioned supernatant for siderophore assays and to identify the additional biochemical requirements of *eqb* NRPS product production on supplementation with 10 μM salicylate (Sigma) (CDMs). To measure the influence of free cation concentration on the growth of wild-type and mutant *S. equi* strains, THA was supplemented with 2 mM NTA (Sigma).

### Identification of *S. equi-*restricted genes

The Artemis Comparison Tool (Carver *et al*., 2005) was used to view blastn alignments of the publicly available sequence data from the *S. equi* and *S. zooepidemicus* genome sequencing projects (http://www.sanger.ac.uk). Comparisons of predicted CDSs with Uniprot were performed using blastp and fasta. Southern blot and PCR analyses were used to determine the genome location, circularization and prevalence of ICE*Se2* across 18 strains of *S. equi* and 73 strains of *S. zooepidemicus* (Table S1). Diverse strains were selected based on their SeM allele (Kelly *et al*., 2006) and RNA intergenic spacer/M protein subtype (Newton *et al*., 2008) respectively.

### Allelic replacement

Internal gene deletions were introduced into *S. equi* 4047 through an allelic replacement strategy previously described for the production of a Δ*prtM* mutant (Hamilton *et al*., 2006). Table S1 shows primers used and details internal frame deletions in the construction of plasmids pG*eqbA*Δ, pG*eqbE*Δ, pG*eqbHIJ*Δ, pG*eqbKL*Δ and pG*ftsB*Δ. The Δ*eqbAE* double deletion mutant was generated by transformation of *S. equi* strain Δ*eqbE* with the pGeqbAΔ plasmid followed by recombination and selection as described previously. Similarly, for other multiple deletion strains, *eqbA* was always the last gene to be deleted. PCR and sequencing were used to confirm the presence of gene deletions.

### RNA isolation

Total RNA was extracted from bacteria using RNAprotect, RNeasy and DNase Kits according to the manufacturer's instructions (Qiagen). Bacteria grown to mid-log phase were pelleted by centrifugation for 10 min at 5000 *g*. The pellet was re-suspended in 200 μl of TE buffer (10 mM Tris-Cl, 1 mM EDTA pH 8.0) containing 3 mg of lysozyme (Sigma) and 500 U mutanolysin (Sigma). After incubation at room temperature for 45 min with repeated vortexing, 700 μl of RLT buffer containing β-mercaptoethanol was added, and the tube was vortexed. The mixture was transferred to a 2 ml reaction tube containing 0.05 g of 100-μm-diameter acid-washed glass beads (Sigma) and vortexed for 5 min. The mixture was then centrifuged and total RNA was extracted from the supernatant using an RNeasy Midi Kit. Five micrograms of recovered RNA was treated with DNase to remove any contaminating DNA followed by clean-up on an RNeasy mini column. The quantity and purity of RNA was determined by optical density measurements at 260 and 280 nm using a NanoDrop® ND1000 spectrophotometer (NanoDrop Technologies).

### Reverse transcription and quantitative real-time PCR

Transcriptional regulation of the NRPS gene (*eqbE*) by the putative repressor (EqbA) was assessed through the comparison of *eqbE* transcript levels in the Δ*eqbA* and parent 4047 *S. equi* strains grown to mid-log phase in THB. A quantitative two-step reverse transcription (RT) PCR procedure was used to analyse levels of *eqbE* gene transcription relative to the housekeeping gene *gyrA*. RT was performed using the Verso cDNA kit (Abgene). The RT reaction mixture (20 μl) contained 96 ng of total RNA, 2 μM gene-specific antisense primer (Table S1), 500 μM dNTP mix, 16 U RNase inhibitor (RNASIN, Promega), 1× cDNA synthesis buffer, 1 μl of RT enhancer and 1 μl of Verso enzyme mix. RT was performed at 50°C for 30 min and terminated by heating to 95°C for 2 min. Quantitative real-time PCR (QPCR) was performed with a Techne Quantica instrument and data were analysed using Quansoft software (Techne). For the QPCR, 6 μl of RT reaction mixture diluted 1/1000 was mixed with 0.3 μM forward and reverse primers (Table S1), 1× ABsolute QPCR SYBR green mix (Abgene) and 40 nM ROX in a total volume of 20 μl and subjected to thermocycling at 95°C for 15 min, followed by 40 cycles of 95°C for 15 s and 60°C for 1 min. Dissociation curves were analysed, following a final ramp step from 60°C to 90°C with reads at 0.5°C increments, to rule out non-specific amplification. No-template-negative controls and reverse transcriptase-negative controls were included to confirm the absence of contaminating DNA from RNA samples. Standard curves (Cp versus log gene copy number) with an efficiency of 1.9 (*R*^2^ = 0.999) were generated from gDNA for each target gene and used to calculate gene copy number in cDNA samples generated from three independent RNA extractions. *eqbE* gene copy number was normalized to *gyrA* reference gene copy number to correct for differences in the amount of starting material. Data were expressed as fold increase in normalized *eqbE* transcript level in the Δ*eqbA* mutant strain relative to the wild-type 4047 strain.

### Streptonigrin sensitivity

The minimum inhibitory concentration (MIC) of streptonigrin and erythromycin was determined following overnight incubation of bacterial strains in a standard 96-well microtitre plate assay. One hundred microlitres of re-suspended colony material corresponding to 10^4^ colony-forming units (cfu) ml^−1^ in THB, CDMs, MM or conditioned THB/CDMs/MM was added to 100 μl of THB supplemented with streptonigrin (concentration ranging from 2 μM to 0.5 nM through twofold serial dilutions) in duplicate wells of a microtitre plate. Sensitivity to erythromycin was determined using the same procedure (0.5 μg ml^−1^ to 0.5 ng ml^−1^ erythromycin).

### ^55^FeCl_3_ accumulation

Wild-type and mutant *S. equi* strains were depleted of iron by growth overnight on THA supplemented with 4 mM NTA. Colony material from these plates was re-suspended in PBS with the volume adjusted to give an optical density at 600 nm of 1.0. Half millilitre of this bacterial suspension was added to 0.5 ml of THB supplemented with 2.0 μCi ml^−1 55^FeCl_3_ (Promega) and incubated at 37°C with 5% CO_2_. After 3 h, cell growth was monitored through measurement of optical density and a volume of cells equivalent to 6 × 10^7^ cfu was collected in triplicate wells of a 96-well UniFilter® GF/CR (Perkin Elmer) with a Packard FilterMate™ Harvester. Cells were washed on the filter with 10 mM NTA and then methanol. The filter was dried for 1 h at room temperature prior to the addition of 20 μl of Microscint 20 (Packard). Bacterial iron accumulation was measured by liquid scintillation counting on a TopCount NXT™ microplate counter (Packard).

A modification of this method was used to analyse the accumulation of ^55^FeCl_3_ in the *S. equi* mutant strain Δ*eqbAE* when cross-fed with filter-sterilized culture supernatant from wild-type, Δ*eqbAE*, Δ*eqbHIJA* or Δ*eqbA* strains grown to stationary phase overnight. Bacteria were re-suspended in 1 ml of 2× THB supplemented with 2.0 μCi ml^−1 55^FeCl_3_ to an optical density at 600 nm of 0.2 and mixed with an equal volume of filter-sterilized culture supernatant. Cells were incubated at 37°C with 5% CO_2_ until they reached an optical density at 600 nm of 0.5 and then a volume of cells equivalent to 1 × 10^8^ cfu was collected in triplicate wells of a 96-well UniFilter® GF/CR and processed as described above.

### Complementation of *ΔeqbA* and *ΔeqbE*

Full-length copies of the *eqbA* or *eqbE* genes were cloned into the plasmid pGhost9 under the control of the *eqbA* and *eqbB* promoters using primers listed in Table S1 to generate constructs pGpA*eqbA* and pGpB*eqbE* respectively. The Δ*eqbA* and Δ*eqbAE* mutant strains were transformed with pGpA*eqbA*, pGpB*eqbE* or pGhost9 control plasmid. Transformants were selected with erythromycin and grown at 28°C, which permits extrachromosomal replication of pGhost9, for 48 h to enable comparison of transformant phenotypes.

### LC-MS analyses of culture supernatants

Comparative metabolic profiling of the Δ*eqbA*, Δ*eqbAE* and Δ*eqbHIJA* strains was performed using LC-MS. Culture supernatants (50 ml) were extracted with ethyl acetate (3 × 50 ml) and both the combined organic extract and the residual culture supernatant were concentrated to dryness and re-dissolved in 20% acetonitrile/0.1% formic acid in water (1 ml). Each sample was split into two equal portions and ferric chloride was added to one portion of each sample to a final concentration of 5 mM. Fifty microlitres of each sample was analysed on an Agilent Zorbax C-18 reverse phase column (150 × 4.6 mm) connected to an Agilent 1100 HPLC instrument. The outflow was transferred via a splitter (90% to waste, 10% to mass spectrometer) to a Bruker HCT+ mass spectrometer, equipped with an electrospray source, operating in positive ion mode. The column was eluted as follows (mobile phase A: water with 0.1% formic acid, mobile phase B: acetonitrile with 0.1% formic acid): 100% mobile phase A for 5 min; a gradient from 100% mobile phase A to 100% mobile phase B over 25 min; 100% mobile phase B for 5 min; a gradient from 100% mobile phase B to 100% mobile phase A over 5 min; 100% mobile phase A for 5 min.

### Chrome azurol S assay

Iron-chelating activity in supernatant samples was monitored by the chrome azurol S assay (Schwyn and Neilands, 1987). Bacteria were grown overnight in 10 ml of CDMs to stationary phase and then filter-sterilized through a 0.22 μm filter (Millipore) to recover supernatant. Supernatant (0.5 ml) was mixed with an equal volume of CAS assay solution and change in absorbance at 630 nm was measured over time. Desferoxamine (Sigma) was included as a standard.

### Overexpression and purification of EqbA

The *S. equi eqbA* gene was amplified by PCR from 4047 chromosomal DNA with Phusion polymerase (NEB), primer ZM329 and primer ZM330 (Table S1). The PCR product was cloned into the BamHI/EcoRI sites of the pGEX-3X vector (GE Healthcare). The resulting construct, pGEX-*eqbA*, contains an N-terminal fusion of *eqbA* to a glutathione S-transferase (GST) tag driven by a tac promoter. DH10B *E. coli* cells harbouring the plasmid pGEX-*eqbA* were grown at 37°C with shaking (220 r.p.m.) in 2× YT containing 50 μg ml^−1^ ampicillin. Once the cells reached an optical density at 600 nm of 0.6, 1 mM isopropyl-β-D-thiogalactopyranoside (IPTG) was added to the medium and the culture was incubated for 4 h at 28°C with shaking (220 r.p.m.). Cells were then harvested and lysed by sonication, and the GST–EqbA fusion was purified over glutathione sepharose 4B beads according to the supplier's protocol (GE Healthcare). Recombinant EqbA was cleaved from the beads by Factor Xa protease cleavage in 50 mM Tris-HCl (pH 7.5), 150 mM NaCl and 1 mM CaCl_2_ according to the supplier's protocol (GE Healthcare). The purified EqbA protein appeared homogenous by SDS-polyacrylamide gel electrophoresis and Coomassie blue staining.

### Electrophoretic mobility shift assay

5′ Biotin end-labelled DNA fragments A, B and C containing the upstream region of the *eqb* operon were amplified from the *S. equi* 4047 chromosome using the primers indicated in Table S1. Fragment A contains the −237 to −11 bp region upstream of the *eqb* operon (P_eqb_), while fragments B and C contain regions −237 to −73 bp and −237 to −135 bp, respectively, and lack the DNA palindrome of P_eqb_ (−73 to −38 bp). The PCR products were purified from a 1.5% agarose gel using a gel extraction kit (Qiagen). Binding reactions (20 μl) were carried out at room temperature for 20 min in 22.5 mM Tris-HCl (pH 7.5), 67.5 mM KCl, 5 mM MgCl_2_, 1.45 mM dithiothreiotol (DTT), 5% glycerol and 1 μg of dIdC. Biotin-labelled target-DNA (20 fmol) was mixed with 300 pmol of EqbA with or without the addition of 125 μM freshly prepared FeSO_4_, ZnSO_4_, MnCl_2_, CuSO_4_ or FeC_6_H_5_O_7_. To remove divalent metal ion from the purified EqbA, the protein was incubated with 2 mM EDTA for 2.5 h with mixing at room temperature and then buffer exchanged against 20 mM Tris-HCl (pH 7.5), 50 mM KCl, 1 mM DTT using a vivaspin500 column (Sartorius). Samples were analysed by electrophoresis at 200 V on a 5.5% non-denaturing polyacrylamide gel containing 2.5% glycerol in 0.5× Tris-borate-EDTA buffer. Transfer to a positively charged nylon membrane (Roche) and the subsequent detection of biotin-labelled DNA by chemiluminescence were performed using the LightShift® Chemiluminescent EMSA Kit (Pierce) according to the manufacturer's instructions. Kit controls were used to analyse the binding of the P_eqb_ fragment and EqbA to an alternative protein extract and target DNA respectively.

### Reconstitution of the equibactin NRPS in *E. coli*

Novagen expression plasmids with compatible replicons were used for the coexpression of *eqbBCD*, *eqbMN*, *eqbF*, *eqbG* (multicystronic) and *eqbE* from T7 promoters in *E. coli* strain BL21(DE3). DNA fragments containing *eqbBCD*, *eqbMN*, *eqbF*, *eqbG* and *eqbE* genes were amplified by PCR from 4047 chromosomal DNA with Phusion polymerase (NEB), using primer pairs indicated in Table S1. *eqbBCD* was cloned into multiple cloning site (MCS)-1 of pACYCDuet-1 using NcoI/BamHI to generate pACYC-BCD. *eqbMN* was cloned into MCS-2 of pACYC-BCD using BglII/AatII to generate pACYC-BCD-MN (chloramphenicol resistant). *eqbF* was cloned into MCS-1 of pCDFDuet-1 using NcoI/BamHI to generate pCDF-F. *eqbG* was cloned into MCS-2 of pCDF-F using FseI/AatII to generate pCDF-F-G (spectinomycin resistant). *eqbE* was cloned into pET21a using NheI/BamHI to generate pET21a-E (ampicillin resistant).

pACYC-BCD-MN, pCDF-F-G and pET21a-E were introduced to BL21(DE3) via electroporation. The resultant *E. coli* strain was grown in Luria–Bertani (LB) broth media or a MM containing: 1.5 mg ml^−1^ KH_2_PO_4_, 4.34 mg ml^−1^ K_2_HPO_4_, 0.4 mg ml^−1^ (NH_4_)_2_SO_4_, 0.22 mg ml^−1^ MgSO_4_·7H_2_O, 5 mg ml^−1^ glucose, 24.5 mg ml^−1^ FeC_6_H_5_O_7_, 2.76 mg ml^−1^ ZnSO_4_·7H_2_O, 1 mg ml^−1^ CaCl_2_, 2 mg ml^−1^ Na_2_MoO_4_·2H_2_O, 1.21 mg ml^−1^ CuSO_4_ and 0.5 mg ml^−1^ H_3_BO_3_. Medium was supplemented with 50 μg ml^−1^ ampicillin, 50 μg ml^−1^ spectinomycin and 34 μg ml^−1^ chloramphenicol. Cultures were inoculated 4% (v/v) with a starter culture and growth was carried out at 37°C on a rotary shaker (220 r.p.m.) to an optical density at 600 nm between 0.6 and 0.8. IPTG (0.4 mM) was added together with 1 mM salicylate and the culture was incubated for a further 20 h at 28°C with shaking (220 r.p.m.). Supernatant was filter-sterilized and collected for LC-MS analysis or to determine its effect on streptonigrin sensitivity following cross-feeding to the Δ*eqbAE* strain as described above.

Additional combinations of plasmids were constructed in order to assess the contribution of EqbE, EqbG, EqbM and EqbN to the biosynthesis of the *eqb* NRPS product(s) in *E. coli* and also to provide appropriate negative controls for this system of production. *eqbM* and *eqbN* were cloned separately into MCS-2 of pACYC-BCD using BglII/AatII to generate pACYC-BCD-M and pACYC-BCD-N respectively (Table S1). Two *E. coli* strains lacking either EqbE or EqbG contained pACYC-BCD-MN, pCDF-F-G, pET21a or pACYC-BCD-MN, pCDF-F, pET21a-E respectively. Another two strains lacking EqbM or EqbN contained pACYC-BCD-N, pCDF-F-G, pET21a-E or pACYC-BCD-M, pCDF-F-G, pET21a-E respectively.
