# Supplementary material for: A novel streptococcal integrative conjugative element involved in iron acquisition
Source: Mol Microbiol. 2008 Oct 22;70(5):1274–92. doi: 10.1111/j.1365-2958.2008.06481.x (PMC3672683; doi:10.1111/j.1365-2958.2008.06481.x)
Supplement: Supplementary file 1 [file mmi0070-1274-SD1.pdf]

A novel streptococcal integrative conjugative element involved in iron acquisition

Zoe Heather[1], Matthew T. G. Holden[2], Karen F. Steward[1], Julian Parkhill[2], Lijiang Song[3], Gregory L. Challis[3], Carl Robinson[1], Nicholas Davis-Poynter[1*] and Andrew S. Waller[1‡]

[1]Centre for Preventive Medicine, Animal Health Trust, Lanwades Park, Kentford, Newmarket, Suffolk, CB8 7UU, UK.
[2] Wellcome Trust Sanger Institute, Wellcome Trust Genome Campus, Hinxton, Cambridge, CB10 1SA, UK.
[3] Department of Chemistry, University of Warwick, Coventry, CV4 7AL, UK.
* Present address. Sir Albert Sakzewski Virus Research Centre, Royal Children's Hospital and Clinical Medical Virology Centre, University of Queensland, Australia
[‡] To whom correspondence should be addressed. Email: andrew.waller@aht.org.uk. Tel: 08700 502424. Fax: 08700 502425.

Supplementary Table S1. Oligonucleotides used in this Study (restriction sites underlined).

Primer	Sequence (5'-3')	Purpose
ZM236	TTTTTTCTTCTTCCCACTGGC	Left flank of ICE*Se2* (498bp) and circular form of ICE*Se2* (~537bp)
ZM343	TTGGGATGACCATGGGATAC	Left flank of ICE*Se2* (498bp)
ZM235	ATTGGGAACACCTTGCAAGG	Right flank of ICE*Se2* (1590bp) and circular form of ICE*Se2* (~537bp)
ZM344	TGTTGGTGTTCCTGGGATTC	Right flank of ICE*Se2* (1590bp)
ZM233	CGTGATTTGAGAGTAGGGAC	*eqbN* probe for Southern blots (317bp)
ZM234	TGTAACGAAGTCGCTACTGC	
EqbE f	AAGATATAGCAGCATCGTATCG	Sense primer QRT-PCR of *eqbE* (130bp)
EqbE r	TCTAAATCTCTATTAAATAGCGGTATATTG	Anti-sense primer
GyrA f	AAGGCGGGATTCCTAAAATC	Sense primer QRT-PCR of *gyrA* (143bp)
GyrA r	GATAAGTAAGCCCTCTAAAATGTG	Anti-sense primer
ZM94	GAGGTCGACGTCACAACAAGACTCTTCCC	*eqbE* deletion (amino acids 4 to 2017)
ZM95	GACGATATCATTATAGAAGGGAGTTTATGATG	
ZM96	GACGATATCAAGTTCCATTCCAAAATCCTCC	
ZM97	GACGAATTCTCGGAATCGCTGAAGGATTG	
ZM123	GACGAATTCACTTTTACAACCGGACAGTTC	*eqbA* deletion (amino acids 2 to 124)
ZM124	GACGATATCATAAACTCATCTAACTTATCCCTTC	
ZM125	GACGATATCCAAGATATAAGAGAAAGAACATGC	
ZM126	GACGTCGACCCATTCGGATTTGGTAACCC	
ZM199	GACGCGGAGGAATTCTTGCTGTAG	*eqbHIJ* deletion (amino acids 3-*eqbH* to 419-*eqbJ*)
ZM198	GACGACGATATCGCTCATTATTATTCTCCTCTGTC	
ZM178	GACGACGATATCGAAGGTATGCTTGCAGTGTC	
ZM179	GACGACGTCGACAACATCGCAAGAGCCATCTC	
ZM162	GACGAATTCGGGTCTTAGAACTTTAGAGG	*eqbKL* deletion (amino acids 3-*eqbK* to 572-*eqbL*)
ZM163	GACGATATCGCTCATGTAACTTCTCCACC	
ZM164	GACGATATCAGGGGTAAAACAGAGGACTG	
ZM165	GGGGTCGACCTGGCATACAAATAACGTCTCC	
ZM180	GACGACGAATTCTTACCTCAGCTGCAAGAAGC	*ftsB* deletion (amino acids 23 to 304)
ZM181	GACGACGATATCACTTGAGCAGGCGACTAATG	
ZM182	GACGACGATATCATCCTCTCAACTGGTGCAAG	
ZM183	GACGACGTCGACAGCCTCATTTGAGTGTAGCC	
ZM329	GACGACGGATCCTTATGAATAAAATATATCATAAGG	Construction of pGEX-EqbA
ZM330	GACGACGAATTCCATGTTCTTTCTCTTATATCTTG	
BIOZM360	AAGATATAAGAGAAAGAACATGC	Amplification of 5' biotin end-labeled target DNA for EMSA
ZM361	TCCATCTGATTATTAATATACTAC	Used with BIOZM360 to amplify 227 bp

		P_{eqb} target DNA A for EMSA
ZM362	TTAATCCTTCTAACATAAAAAGTC	Used with BIOZM360 to amplify 165 bp target DNA B for EMSA
ZM452	CTAAAAGGCTTGAAGAAACTAAG	Used with BIOZM360 to amplify 103 bp target DNA C for EMSA
ZM248	TAGTATCATCCATGGAAGGTGTTTTGATGGG	eqbBCD for cloning pACYC-BCD
ZM249	CACGACGGATCCAGTTCCATTCCAAAATCCTCC	
ZM250	GACGACGACAGATCTTATGTATAATATAGGAGTTTTAGGTTG	eqbMN for cloning pACYC-BCD-MN
ZM251	GTAGGTAGCGACGTCCAGCCAATAAAACCCACTAATG	
ZM311	GACGACGACAGATCTAATGGATATGAGTTATTTGAATTTA	eqbN for cloning pACYC-BCD-N
ZM312	GTAGGTAGCGACGTCTTATAGATTAAGTGGTGATGTGC	eqbM for cloning pACYC-BCD-M
ZM252	GACGACGACCCATGGTTATGATGAAAAAAGTTATTATATGTGG	eqbF for cloning pCDF-F
ZM253	GACGGATCCCAAGACCTTTTCTGTACAACTC	
ZM254d	GACGACGACGGCCGGCCTCATGAAAATAGAGAATAAATTAATAGAG	eqbG for cloning pCDF-F-G and pCDF-G
ZM255	CACTACGCCGACGTCTTATTGCTCATTATTATTCTCCTC	
ZM240	GACGACGACGCTAGCATGGAACTTAACAATATAAAAGAAAG	eqbE for cloning pET21a-E
ZM241	GACGACGGATCCTTTTTTCATCATAAACTCCCTTC	
ZM446	GACGACGAATTCGTAGTATAACAATAATAAGAATGTTG	pA*eqbA* for cloning pGpA*eqbA*
ZM447	GACGACGACGTCGACTCTTTCTCTTATATCTTGTCTTC	
ZM442	GACGACGACCCCGGGCAAGATATAAGAGAAAGAACATGC	Amplification of pB for recombinant PCR
ZM443	CTTTCTTTTATATTGTTAAGTTCCATCAAAACACCTTCCATCTGATTATTAATATAC	
ZM444	GTATATTAATAATCAGATGGAAGGTGTTTTGATGGAACTTAACAATATAAAAGAAAG	Amplification of *eqbE* N terminal region for recombinant PCR. ZM442/ZM445 used to amplify recombinant pB*eqbE* product. HindIII/SalI digestion used to move the remaining region of *eqbE* from pET21a-E to construct pGpB*eqbE*
ZM445	GTTCTAAGCATGGGATGTCTG	

Supplementary Table S2. BlASTP/FASTA analysis of the *eqb* cluster.

	Match by FASTA/BLASTP analysis					
***S. equi* CDS**	**CDS**	**Amino acid sequence identity %**	**Function**	**Organism**	**Accession no.**	**Reference**
EqbA	gbs1749	42	Putative repressor	*Streptococcus agalactiae serotype III*	Q8E3K9	Glaser *et al.*, 2002
	CKL_1082	40	Putative repressor	*Clostridium kluyveri*	A5N743	Seedorf *et al.*, 2008
	MntR	33	Manganese-dependant repressor	*Bacillus subtilis*	P54512	Que and Helmann, 2000
	DtxR	28	Iron-dependant repressor	*Corynebacterium diphtheriae*	P33120	Qiu *et al.*, 1996
EqbB	CKL_1503	52	Thioesterase	*Clostridium kluyveri*	A5N8B4	Seedorf *et al.*, 2008
	PchC	34	Thioesterase (pyochelin biosythesis)	*Pseudomonas aeruginosa*	P72176	Serino *et al.*, 1997
	ybtT	31	Thioesterase (yersiniabactin biosynthesis)	*Yersinia pestis*	Q56949	Bearden *et al.*, 1997
EqbC	GSP	27	4'-phospho-pantetheinyl-transferase	*Bacillus migulanus*	P40683	Lambalot *et al.*, 1996
	Sfp	25	4'-phospho-pantetheinyl-transferase	*Bacillus subtilis*	P39135	Quadri *et al.*, 1998
	CKL_1523	23	Putative 4'-phospho-pantetheinyl- transferase (Sfp)	*Clostridium kluyveri*	A5N8D4	Seedorf *et al.*, 2008
EqbD	CKL_1504	55	Putative aryl AMP ligase	*Clostridium kluyveri*	A5N8B5	Seedorf *et al.*, 2008
	YbtE	42	Salicyl-AMP ligase (yersiniabactin biosythesis)	*Yersinia pestis*	Q56950	Gehring *et al.*, 1998a
	DhbE	41	2,3-dihydroxy-benzoate-AMP ligase (bacillibactin biosynthesis)	*Bacillus subtilis*	P40871	May *et al.*, 2002
EqbE	CKL_1505	45	Non-ribosomal peptide synthetase (putative siderophore biosynthesis)	*Clostridium kluyveri*	A5N8B6	Seedorf *et al.*, 2008
	HMWP2	30	Non-ribosomal peptide synthetase (yersiniabactin biosynthesis)	*Yersinia pestis*	Q9Z399	Gehring *et al.*, 1998b
	Irp2	30	Non-ribosomal peptide synthetase (siderophore biosynthesis)	*Photobacterium damselae* subspecies *piscicida*	Q2HQW9	Osorio *et al.*, 2006
EqbF	YbtU	32 (partial)	Thiazoline reductase	*Yersinia pestis*	Q9Z3C6	Gehring *et al.*, 1998a
	CKL_1506	32 (partial)	Putative NRPS reductase	*Clostridium kluyveri*	A5N8B7	Seedorf *et al.*, 2008
	CKL_1510	31 (partial)	Putative NRPS reductase	*Clostridium kluyveri*	A5N8C1	Seedorf *et al.*, 2008
	Irp3	26 (partial)	Putative oxidoreductase (siderophore biosynthesis)	*Photobacterium damselae* subspecies *piscicida*	Q2HQW7	Osorio *et al.*, 2006
	PchG	25 (partial)	NADPH-dependent thiazoline reductase (pyochelin biosynthesis)	*Pseudomonas aeruginosa*	Q9HWG5	Reimmann *et al.*, 2001
EqbG	CKL_1511	25	Non-ribosomal peptide	*Clostridium kluyveri*	A5N8C2	Seedorf *et*

			synthetase (putative siderophore biosynthesis)			*al.*, 2008
	HMWP1	25 (partial)	Non-ribosomal peptide/polyketide synthetase (yersiniabactin biosynthesis)	*Yersinia enterocolitica*	O54511	Pelludat *et al.*, 1998
	PchF	29 (partial)	Non-ribosomal peptide synthetase (enantio-pyochelin biosynthesis)	*Pseudomonas fluorescens*	A8U045	Youard *et al.*, 2007
EqbH	Cbei_3098	54	Putative membrane protein	*Clostridium beijerinckii*	A6LY05	
EqbI	Cbei_3097	34	Putative cobalt transport membrane protein	*Clostridium beijerinckii*	A6LY04	
	CKL_1516	24	Putative transporter (membrane protein)	*Clostridium kluyveri*	A5N8C7	Seedorf *et al.*, 2008
EqbJ	Cbei_3096	48	Putative ABC transporter ATPase	*Clostridium beijerinckii*	A6LY03	
	CKL_1517	45	Putative ABC transporter ATPase	*Clostridium kluyveri*	A5N8C8	Seedorf *et al.*, 2008
EqbK	SAS2320	55	Putative ABC transporter (ATPase and permease)	*Staphylococcus aureus*	Q6G6P2	Holden *et al.*, 2004
	Cbei_3094	48	Putative ABC transporter (ATPase and permease)	*Clostridium beijerinckii*	A6LY01	
	CKL_1512	42	Putative ABC transporter (ATPase and permease)	*Clostridium kluyveri*	A5N8C3	Seedorf *et al.*, 2008
	YbtP	29 (no match in N term 69 aa)	Yersiniabactin importer	*Yersinia pestis*	Q9ZG01	Fetherston *et al.*, 1999
	YbtQ	29 (no match in N term 109 aa)	Yersiniabactin importer	*Yersinia pestis*	Q9Z375	Fetherston *et al.*, 1999
EqbL	SAS2319	57	Putative ABC transporter	*Staphylococcus aureus*	Q6G6P3	Holden *et al.*, 2004
	Cbei_3093	46	Putative ABC transporter (ATPase and permease)	*Clostridium beijerinckii*	A6LY00	
	CKL_1513	40	Putative ABC transporter (ATPase and permease)	*Clostridium kluyveri*	A5N8C4	Seedorf *et al.*, 2008
	YbtP	28 (no match in N term 109 aa)	Yersiniabactin importer	*Yersinia pestis*	Q9ZG01	Fetherston *et al.*, 1999
	YbtQ	30 (no match in N term 125 aa)	Yersiniabactin importer	*Yersinia pestis*	Q9Z375	Fetherston *et al.*, 1999
EqbM	CKL_1509	31% (partial)	unknown	*Clostridium kluyveri*	A5N8C0	Seedorf *et al.*, 2008
EqbN	Csac_2163	31% (partial)	unknown	*Caldicellulosiruptor saccharolyticus*	A4XLG0	

Supplementary Figure S1. Comparison of ICE*Se2* with the conjugative transposons CDTn*2* and CDTn*5* of *C. difficile* Strain 630 (Sebaihia *et al.*, 2006) and Tn*1549* from *E. faecalis* (Garnier *et al.*, 2000) (Displayed using ACT (Carver *et al.*, 2005)).

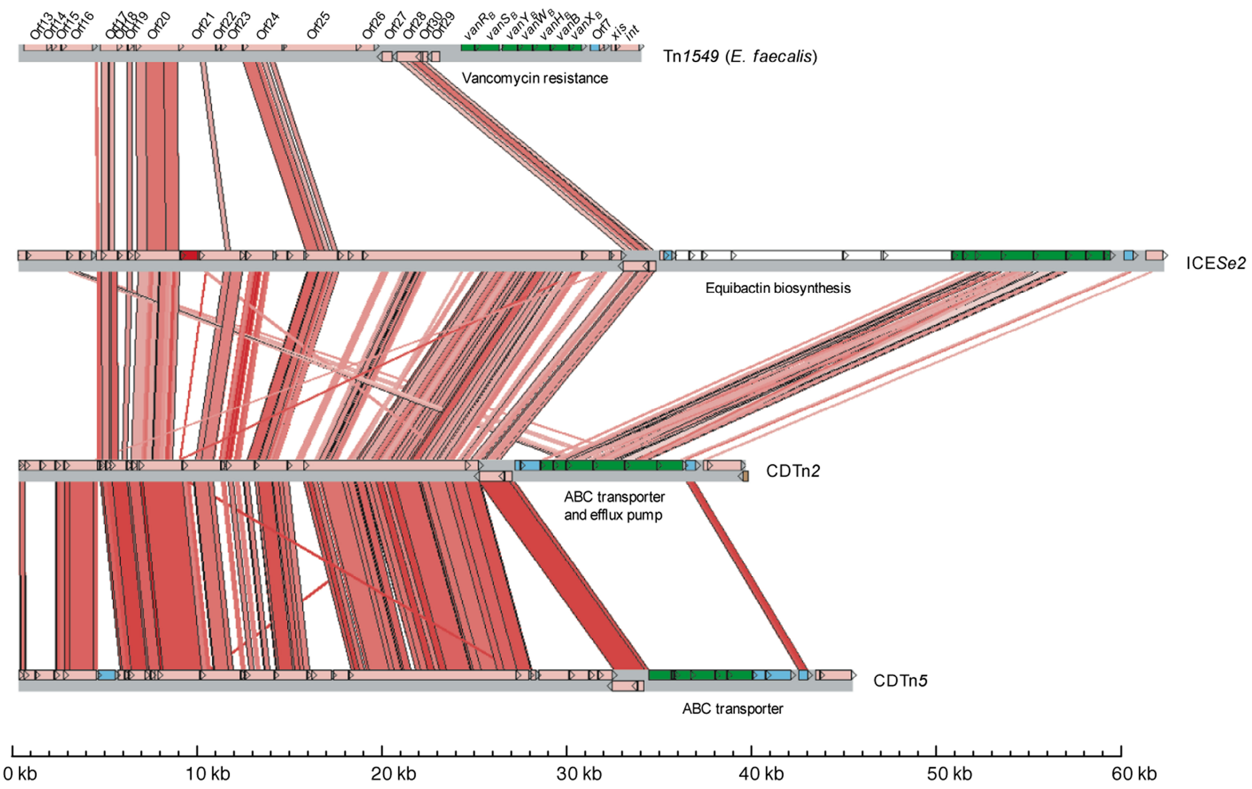

Grey bars represent the forward and reverse strands of DNA. The red lines between the elements represent protein similarity (TBLASTX). CDSs involved in similar function are given the same color: pink, conjugal transfer and integration and excision; blue, regulation; white, secondary metabolism; green, transport; red, accessory function. The gene names of Tn*1549* are shown on top. The genes encoding the equibactin NRPS are shown in white and those encoding the ABC transporters associated with the equibactin locus are shown in green.

Supplementary Figure S2. The influence of gene complementation on colony phenotype in wild-type and mutant *S. equi* strains.

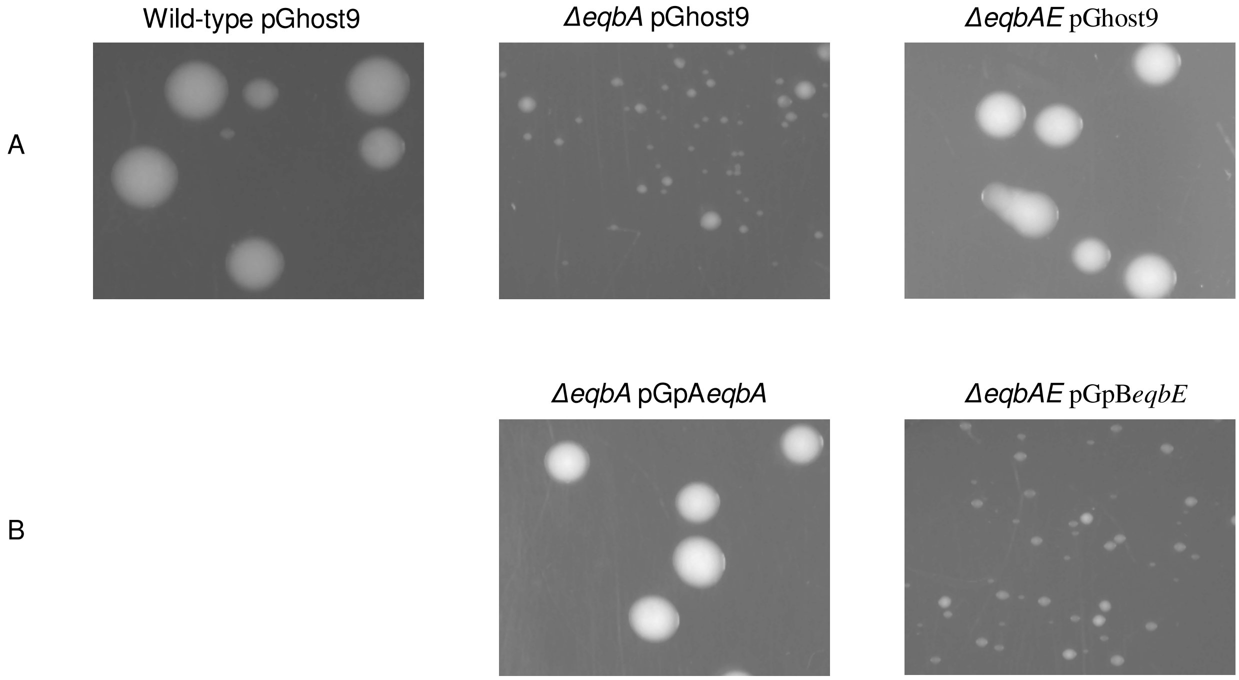

A. Photographs show colonies of wild-type, *ΔeqbA* and *ΔeqbAE* strains carrying the pGhost9 control plasmid grown for 48 hr on THA supplemented with erythromycin. B. Photographs show the change in colony size of the *ΔeqbA* and *ΔeqbAE* mutants complemented with a second copy of *eqbA* under the control of the *eqbA* promoter (pGpA*eqbA*) or a second copy of *eqbE* under the control of the *eqbB* promoter (pGpB*eqbE*), respectively.

Supplementary Figure S3. CLUSTALW alignment of IdeRs.

```
EqbA (S.equi)  MNKIYHKEYVEQNYLENIYTLYLE--RDEVRNIDIVDKLGVARATVTHMLRNLEKKGYIK 58
DtxR (C.dip)   ---MKDLVDTTEMYLRTIYELEEE--GVTPLRARIAERLEQSGPTVSQTVARMERDGLVV 55
MntR (B.sub)   -----MTTPSMEDYIEQIYMLIEE--KGYARVSDIAEALAVHPSSVTKMVQKLDKDEYLI 53
TroR (T.pal)   --MSLVSDIAAENYLKTVVKALARSRRERVGTGELSRLLHVTPGTISTMVKRLEKGGYVQ 58
                          : *:. :     .          :   *     :::  : .:::   : 

EqbA (S.equi)  YGDDKIVRFTSKGRTLAVELYEKHIYLTQVFK-HIGVDEKIAEIEACQIEHIISKDTFN- 116
DtxR (C.dip)   VASDRSLQMTPTGRTLATAVMRKHRLAERLLTDIIGLDINKVHDEACRWEHVMSDEVERR 115
MntR (B.sub)   YEKYRGLVLTSKGKKIGKRLVYRHELLDQFLR-IIGVDEEKIYNDVEGIEHHLSWNSIDR 112
TroR (T.pal)   RTHRLGCTLTRKGAVFGSAVLRKHRLLESFLSQVLCLEAGVVHKEAEMLEHACSDELIDV 118
                       :* .*  :.  :  :*     .:   : ::      :.   **  * :

EqbA (S.equi)  --KIKKYFE-----------------DKI------------------------------- 126
DtxR (C.dip)   LVKVLKDVSRSPFGNPIPGLDELGVGNSDAAVPGTRVIDAATSMPRKVRIVQINEIFQVE 175
MntR (B.sub)   IGDLVQYFE-----------------EDDARKKDLKSIQKKTEHHNQ------------- 142
TroR (T.pal)   IDRYLQYPTRDPHG------------QPIPRKDTLLDLYVEDDVPGV------------- 153
                    :                    :

EqbA (S.equi)  ---------------------------------------------------
DtxR (C.dip)   TDQFTQLLDADIRVGSEVEIVDRDGHITLSHNGKDVELIDDLAHTIRIEEL 226
MntR (B.sub)   ---------------------------------------------------
TroR (T.pal)   ---------------------------------------------------
```

Primary structure alignment of EqbA homologues known to be regulated by Fe^{2+} or Mn^{2+}. Sequence alignment was generated using CLUSTALW. The putative residues involved in the coordination of the regulatory metal are shown in bold. The Helix-Turn-Helix motif is underlined. Sequences used are: DtxR, P33120 (Qiu *et al.*, 1996); MntR, P54512 (Que and Helmann, 2000); TroR, P96120 (Hardham *et al.*, 1997).

References for Supplementary information.

Bearden, S.W., Fetherston, J.D., and Perry, R.D. (1997) Genetic organization of the yersiniabactin biosynthetic region and construction of avirulent mutants in Yersinia pestis. *Infect Immun* **65**: 1659-1668.

Carver, T.J., Rutherford, K.M., Berriman, M., Rajandream, M.A., Barrell, B.G., and Parkhill, J. (2005) ACT: the Artemis Comparison Tool. *Bioinformatics* **21**: 3422-3423.

Fetherston, J.D., Bertolino, V.J., and Perry, R.D. (1999) YbtP and YbtQ: two ABC transporters required for iron uptake in Yersinia pestis. *Mol Microbiol* **32**: 289-299.

Garnier, F., Taourit, S., Glaser, P., Courvalin, P., and Galimand, M. (2000) Characterization of transposon Tn1549, conferring VanB-type resistance in Enterococcus spp. *Microbiology* **146 (Pt 6)**: 1481-1489.

Gehring, A.M., DeMoll, E., Fetherston, J.D., Mori, I., Mayhew, G.F., Blattner, F.R., Walsh, C.T., and Perry, R.D. (1998a) Iron acquisition in plague: modular logic in enzymatic biogenesis of yersiniabactin by Yersinia pestis. *Chem Biol* **5**: 573-586.

Gehring, A.M., Mori, I., Perry, R.D., and Walsh, C.T. (1998b) The nonribosomal peptide synthetase HMWP2 forms a thiazoline ring during biogenesis of yersiniabactin, an iron-chelating virulence factor of Yersinia pestis. *Biochemistry* **37**: 11637-11650.

Glaser, P., Rusniok, C., Buchrieser, C., Chevalier, F., Frangeul, L., Msadek, T., Zouine, M., Couve, E., Lalioui, L., Poyart, C., Trieu-Cuot, P., and Kunst, F. (2002) Genome sequence of Streptococcus agalactiae, a pathogen causing invasive neonatal disease. *Mol Microbiol* **45**: 1499-1513.

Hardham, J.M., Stamm, L.V., Porcella, S.F., Frye, J.G., Barnes, N.Y., Howell, J.K., Mueller, S.L., Radolf, J.D., Weinstock, G.M., and Norris, S.J. (1997) Identification and transcriptional analysis of a Treponema pallidum operon encoding a putative ABC transport system, an iron-activated repressor protein homolog, and a glycolytic pathway enzyme homolog. *Gene* **197**: 47-64.

Holden, M.T., Feil, E.J., Lindsay, J.A., Peacock, S.J., Day, N.P., Enright, M.C., Foster, T.J., Moore, C.E., Hurst, L., Atkin, R., Barron, A., Bason, N., Bentley, S.D., Chillingworth, C., Chillingworth, T., Churcher, C., Clark, L., Corton, C., Cronin, A., Doggett, J., Dowd, L., Feltwell, T., Hance, Z., Harris, B., Hauser, H., Holroyd, S., Jagels, K., James, K.D., Lennard, N., Line, A., Mayes, R., Moule, S., Mungall, K., Ormond, D., Quail, M.A., Rabbinowitsch, E., Rutherford, K., Sanders, M., Sharp, S., Simmonds, M., Stevens, K., Whitehead, S., Barrell, B.G., Spratt, B.G., and Parkhill, J. (2004) Complete genomes of two clinical Staphylococcus aureus strains: evidence for the rapid evolution of virulence and drug resistance. *Proc Natl Acad Sci U S A* **101**: 9786-9791.

Lambalot, R.H., Gehring, A.M., Flugel, R.S., Zuber, P., LaCelle, M., Marahiel, M.A., Reid, R., Khosla, C., and Walsh, C.T. (1996) A new enzyme superfamily - the phosphopantetheinyl transferases. *Chem Biol* **3**: 923-936.

May, J.J., Kessler, N., Marahiel, M.A., and Stubbs, M.T. (2002) Crystal structure of DhbE, an archetype for aryl acid activating domains of modular nonribosomal peptide synthetases. *Proc Natl Acad Sci U S A* **99**: 12120-12125.

Osorio, C.R., Juiz-Rio, S., and Lemos, M.L. (2006) A siderophore biosynthesis gene cluster from the fish pathogen Photobacterium damselae subsp. piscicida is structurally and functionally related to the Yersinia high-pathogenicity island. *Microbiology* **152**: 3327-3341.

Pelludat, C., Rakin, A., Jacobi, C.A., Schubert, S., and Heesemann, J. (1998) The yersiniabactin biosynthetic gene cluster of Yersinia enterocolitica: organization and siderophore-dependent regulation. *J Bacteriol* **180**: 538-546.

Qiu, X., Pohl, E., Holmes, R.K., and Hol, W.G. (1996) High-resolution structure of the diphtheria toxin repressor complexed with cobalt and manganese reveals an SH3-like third domain and suggests a possible role of phosphate as co-corepressor. *Biochemistry* **35**: 12292-12302.

Quadri, L.E., Weinreb, P.H., Lei, M., Nakano, M.M., Zuber, P., and Walsh, C.T. (1998) Characterization of Sfp, a Bacillus subtilis phosphopantetheinyl transferase for peptidyl carrier protein domains in peptide synthetases. *Biochemistry* **37**: 1585-1595.

Que, Q., and Helmann, J.D. (2000) Manganese homeostasis in Bacillus subtilis is regulated by MntR, a bifunctional regulator related to the diphtheria toxin repressor family of proteins. *Mol Microbiol* **35**: 1454-1468.

Reimmann, C., Patel, H.M., Serino, L., Barone, M., Walsh, C.T., and Haas, D. (2001) Essential PchG-dependent reduction in pyochelin biosynthesis of Pseudomonas aeruginosa. *J Bacteriol* **183**: 813-820.

Sebaihia, M., Wren, B.W., Mullany, P., Fairweather, N.F., Minton, N., Stabler, R., Thomson, N.R., Roberts, A.P., Cerdeno-Tarraga, A.M., Wang, H., Holden, M.T., Wright, A., Churcher, C., Quail, M.A., Baker, S., Bason, N., Brooks, K., Chillingworth, T., Cronin, A., Davis, P., Dowd, L., Fraser, A., Feltwell, T., Hance, Z., Holroyd, S., Jagels, K., Moule, S., Mungall, K., Price, C., Rabbinowitsch, E., Sharp, S., Simmonds, M., Stevens, K., Unwin, L., Whithead, S., Dupuy, B., Dougan, G., Barrell, B., and Parkhill, J. (2006) The multidrug-resistant human pathogen Clostridium difficile has a highly mobile, mosaic genome. *Nat Genet* **38**: 779-786.

Seedorf, H., Fricke, W.F., Veith, B., Bruggemann, H., Liesegang, H., Strittmatter, A., Miethke, M., Buckel, W., Hinderberger, J., Li, F., Hagemeier, C., Thauer, R.K., and Gottschalk, G. (2008) The genome of Clostridium kluyveri, a strict anaerobe with unique metabolic features. *Proc Natl Acad Sci U S A* **105**: 2128-2133.

Serino, L., Reimmann, C., Visca, P., Beyeler, M., Chiesa, V.D., and Haas, D. (1997) Biosynthesis of pyochelin and dihydroaeruginoic acid requires the iron-regulated pchDCBA operon in Pseudomonas aeruginosa. *J Bacteriol* **179**: 248-257.

Youard, Z.A., Mislin, G.L., Majcherczyk, P.A., Schalk, I.J., and Reimmann, C. (2007) Pseudomonas fluorescens CHA0 produces enantio-pyochelin, the optical antipode of the Pseudomonas aeruginosa siderophore pyochelin. *J Biol Chem* **282**: 35546-35553.
